# Effects of a Four-Week High-Dosage Zinc Oxide Supplemented Diet on Commensal *Escherichia coli* of Weaned Pigs

**DOI:** 10.3389/fmicb.2019.02734

**Published:** 2019-11-28

**Authors:** Vanessa C. Johanns, Fereshteh Ghazisaeedi, Lennard Epping, Torsten Semmler, Antina Lübke-Becker, Yvonne Pfeifer, Astrid Bethe, Inga Eichhorn, Roswitha Merle, Birgit Walther, Lothar H. Wieler

**Affiliations:** ^1^Advanced Light and Electron Microscopy (ZBS-4), Robert Koch Institute, Berlin, Germany; ^2^Institute of Microbiology and Epizootics, Centre for Infection Medicine, Freie Universität Berlin, Berlin, Germany; ^3^Microbial Genomics (NG1), Robert Koch Institute, Berlin, Germany; ^4^Nosocomial Pathogens and Antibiotic Resistances, Robert Koch Institute, Wernigerode, Germany; ^5^Institute for Veterinary Epidemiology and Biostatistics, Freie Universität Berlin, Berlin, Germany; ^6^Robert Koch Institute, Berlin, Germany

**Keywords:** *Escherichia coli*, zinc, antimicrobial resistance, pig, heavy metal tolerance

## Abstract

Strategies to reduce economic losses associated with post-weaning diarrhea in pig farming include high-level dietary zinc oxide supplementation. However, excessive usage of zinc oxide in the pig production sector was found to be associated with accumulation of multidrug resistant bacteria in these animals, presenting an environmental burden through contaminated manure. Here we report on zinc tolerance among a random selection of intestinal *Escherichia coli* comprising of different antibiotic resistance phenotypes and sampling sites isolated during a controlled feeding trial from 16 weaned piglets: In total, 179 isolates from “pigs fed with high zinc concentrations” (high zinc group, [HZG]: *n* = 99) and a corresponding “control group” ([CG]: *n* = 80) were investigated with regard to zinc tolerance, antimicrobial- and biocide susceptibilities by determining minimum inhibitory concentrations (MICs). In addition, *in silico* whole genome screening (WGSc) for antibiotic resistance genes (ARGs) as well as biocide- and heavy metal tolerance genes was performed using an in-house BLAST-based pipeline. Overall, porcine *E. coli* isolates showed three different ZnCl_2_ MICs: 128 μg/ml (HZG, 2%; CG, 6%), 256 μg/ml (HZG, 64%; CG, 91%) and 512 μg/ml ZnCl_2_ (HZG, 34%, CG, 3%), a unimodal distribution most likely reflecting natural differences in zinc tolerance associated with different genetic lineages. However, a selective impact of the zinc-rich supplemented diet seems to be reasonable, since the linear mixed regression model revealed a statistically significant association between “higher” ZnCl_2_ MICs and isolates representing the HZG as well as “lower ZnCl_2_ MICs” with isolates of the CG (*p* = 0.005). None of the zinc chloride MICs was associated with a particular antibiotic-, heavy metal- or biocide- tolerance/resistance phenotype. Isolates expressing the 512 μg/ml MIC were either positive for ARGs conferring resistance to aminoglycosides, tetracycline and sulfamethoxazole-trimethoprim, or harbored no ARGs at all. Moreover, WGSc revealed a ubiquitous presence of zinc homeostasis and – detoxification genes, including *zit*B, *znt*A, and *pit*. In conclusion, we provide evidence that zinc-rich supplementation of pig feed selects for more zinc tolerant *E. coli*, including isolates harboring ARGs and biocide- and heavy metal tolerance genes – a putative selective advantage considering substances and antibiotics currently used in industrial pork production systems.

## Introduction

Enterotoxigenic *Escherichia coli* (ETEC) are commonly associated with post-weaning diarrhea (PWD) in piglets, a disease causing serious losses in the pig industry worldwide (Fairbrother et al., 2005; Rhouma et al., 2017a). Currently, different strategies are utilized to reduce ETEC-associated economic costs in pig farming, including oral colistin sulfate treatment in some regions of the world (Rhouma et al., 2017b), vaccination (Moon and Bunn, 1993; Blázquez et al., 2018) and probiotics (Li et al., 2018; Yan et al., 2018). In addition, high-level dietary zinc oxide supplementation is used against PWD in the pig production sector (Fairbrother et al., 2005; Vahjen et al., 2011; Bednorz et al., 2013; Starke et al., 2014; Pieper et al., 2015; Kloubert et al., 2018).

However, the effects of zinc-rich diets on porcine intestinal bacterial populations, especially *E. coli*, are not fully understood yet. Zinc is the second most abundant transition metal in most phyla and generally considered as essential for life. Together with copper, it is an important trace element required for hormone function, reproduction, vitamin synthesis, enzyme formation and it promotes a strong immune system function (Yu et al., 2017). Both metals are usually added to animal feed in amounts necessary for physiological body function (Yazdankhah et al., 2014; Yu et al., 2017).

As a divalent cation (Zn^2+^), zinc plays an important role as a catalytic and structural cofactor in virtually all aspects of cell metabolism (Vallee and Auld, 1990). Keeping a balanced intracellular zinc homeostasis is a prerequisite for mammals and most bacterial species (Nies and Grass, 2009). Therefore, zinc quantities within cells are highly regulated, as zinc deprivation hinders bacterial growth, while an excess of zinc could be toxic (Gielda and DiRita, 2012). Factors reported to increase zinc tolerance levels in *E. coli* described so far include the cation diffusion facilitator (CDF) ZitB, the P_1__*b*_-type ATPase ZntA and the low-affinity inorganic phosphate transporter Pit (Beard et al., 2000; Grass et al., 2005; Deus et al., 2017; Hoegler and Hecht, 2018).

So far, an increased tolerance toward (trace) metals, including zinc, is clearly linked to genes conferring antibiotic resistance in different bacterial species (Cavaco et al., 2011; Agga et al., 2014; Medardus et al., 2014; Becerra-Castro et al., 2015; Song et al., 2017; van Alen et al., 2018), possibly indicating a worrisome co-selective effect of zinc oxide mass utilization (Seiler and Berendonk, 2012; Bednorz et al., 2013; Yazdankhah et al., 2014; Ciesinski et al., 2018). Consequently, the current anthropogenic contamination of the environment with heavy metals is regarded as a serious problem (Seiler and Berendonk, 2012).

In *E. coli*, an extensive, finely tuned network of efflux pumps, ligands and transcription factors is involved in intracellular osmoadaption and heavy metal detoxification, also warranting maintenance of cellular zinc homeostasis (Hantke, 2005; Nies and Grass, 2009; Porcheron et al., 2013; Watly et al., 2016). Recent studies revealed that zinc tolerance levels differ not only between bacterial species but also within particular species, including *E. coli* of human and avian origin (Deus et al., 2017; Stocks et al., 2019).

The aim of this work is to study the effects of zinc-rich diets on a representatively selected collection of intestinal *E. coli* obtained from post-weaning piglets, considering a putative association of a nutritive zinc oxide excess and (i) phenotypic zinc tolerance levels, (ii) antibiotic- and biocide susceptibility profiles, and (iii) genes involved in antimicrobial resistance, zinc (heavy metal)- and biocide tolerance.

## Materials and Methods

### Sample Size and Isolate Selection

The representative set of *E. coli* isolates investigated here was selected based on a previous feeding trial (Ciesinski et al., 2018) carried out in accordance with the principles of the Basel Declaration following the institutional and national guidelines for the care and use of animals. The protocol was approved by the local state office of occupational health and technical safety “Landesamt für Gesundheit und Soziales, Berlin” (LaGeSo Reg. Nr. 0296/13) as described before (Ciesinski et al., 2018).

Briefly, 32 landrace piglets weaned at day 25 ± 1 were separated in two groups for 4 weeks: the first group of piglets, denoted here as the high-zinc group (HZG) was fed with a diet supplemented with a comparatively high amount of zinc oxide (2,103 mg zinc/kg diet), while the second group served as control. This control group (CG) received a common piglet diet containing a physiologic concentration of zinc oxide (72 mg zinc/kg diet) to avoid trace metal malnutrition (Ciesinski et al., 2018). The trial started with 32 piglets, which were sacrificed mid-trial (38 ± 2 days, *n* = 16) and at the end (52 ± 2 days, *n* = 16).

Here we focus on *E. coli* obtained from samples of pigs sacrificed at the end (*n* = 16; 52 ± 2 days) of the feeding trial only. Altogether, 817 *E. coli* collected from the feces, digesta- and mucosa samples obtained from these final 16 pigs were stored in glycerol stocks at −80° (Ciesinski et al., 2018). Using meta data such as sampling site, feeding group and evaluation of growth on plates containing different antibiotics which were available for all the 817 *E. coli* obtained from this initial approach (Ciesinski et al., 2018) we selected a stratified random sample comprising 179 isolates (Table 1 and Supplementary Table S1).

**TABLE 1:** Distribution of *E. coli* among sampling sites, feeding groups, pigs, ZnCl_2_ MICs and antibiotic resistance profiles.

							**Antibiotic resistance profiles**						
							
													**AMP/**
												**AMP/**	**PIP/**
		**Piglets**	***E. coli***	**ZnCl_2_ MICs (μg/ml)**						**AMP/**	**TET/**	**PIP/**	**TET/**
									
				**128**	**256**	**512**	**suscep.**	**SXT**	**TET**	**PIP**	**SXT**	**TET**	**SXT**
				
**Site**	**Group**	***n***	***n***	***n***	***n***	***n***	***n***	***n***	***n***	***n***	***n***	***n***	***n***
Mucosa	HZG	7	33	1	22	10	18	0	0	0	6	9	0
	CG	8	25	0	25	0	16	0	2	0	1	6	0
Digesta	HZG	8	33	1	17	15	17	0	0	0	8	8	0
	CG	8	25	3	20	2	16	1	1	0	0	3	4
Feces	HZG	8	33	0	24	9	17	0	0	1	6	2	7
	CG	7	30	2	28	0	18	0	0	0	0	9	3

*Distribution of 179 *E. coli* isolates among sampling sites, feeding groups, piglets (*n* = 16), zinc chloride tolerance levels and antibiotic resistance profiles. n, number; HZG, high zinc fed group; CG, control group; suscep., fully susceptible toward antibiotics tested; SXT, sulfamethoxazole-trimethoprim; TET, tetracycline; AMP, ampicillin; PIP, piperacillin.*

### Phenotype Characterization of Porcine *E. coli*

A broth microdilution assay was carried out for biocides and heavy metal salts using microtiter-plates (Merlin, Bornheim-Hersel, Germany) as described before (Deus et al., 2017) including alkyl diaminoethyl glycin hydrochloride [ADH], benzethonium chloride [BEN], benzalkonium chloride [BKC], guanidine chlorhexidine [CHX], acridine compound acriflavine [ACR], copper sulfate [COP], silver nitrate [SIL] and zinc chloride [ZKC]. *E. coli* strains ATCC25922 and ATCC10536 were used for internal quality control. In addition, *E. coli* strain RKI6122 was used as a reference for growth in the presence of 1024 μg ZnCl_2_/ml (Deus et al., 2017).

Antimicrobial susceptibility testing (AST) using the VITEK^®^ 2 system (BioMérieux, Germany; AST card GN38) was performed including amikacin, amoxicillin/clavulanic acid, ampicillin, cephalexin, chloramphenicol, enrofloxacin, gentamicin, marbofloxacin, piperacillin, tetracycline, tobramycin and trimethoprim/sulfamethoxazole according to the standards given by the CLSI VET01-A4 and M100-S21) (Clinical and Laboratory Standards Institute, 2012, 2013).

### Molecular Characterization of *E. coli*

Hundred and seventy nine *E. coli* were sequenced using Illumina MiSeq^®^ 300 bp paired-end whole genome sequencing (WGS) with an obtained coverage of >90X. Plasmid DNA of *E. coli* RKI3099, an isolate representing a frequently occurring genomic background associated with increased zinc tolerance together with antimicrobial resistance (Supplementary Table S1), was isolated using Qiagen Plasmid Mini Kit according to manufacturer’s instructions. The purified plasmid DNA was sequenced using the Pacific Biosciences RS II platform with the P6C4 chemistry in a single flowcell. Pacific Biosciences sequencing was completed by generating a sequencing library from 5 to 20 kb using standard methods. PacBio raw data and the Illumina short reads were hybrid assembled using unicycler v4.4 (Wick et al., 2017). Adapter-trimmed reads were used for *de novo* assembly into contiguous sequences (contigs) and subsequently into scaffolds using SPAdes v3.11. All draft genomes were annotated using Prokka (Seemann, 2014).

In previous studies, factors have been described as being capable to confer elevated zinc tolerance levels in *E. coli* (Grass et al., 2005; Deus et al., 2017; Vidhyaparkavi et al., 2017; Stocks et al., 2019). Consequently, we analyzed the presence or absence of a broad set of genes involved in zinc homeostasis (*n* = 35) including zinc-binding metalloenzymes (*n* = 69) (Supplementary Table S2).

Since co-selection of antibiotic- and metal resistance is an issue of utmost importance and metal resistance genes are often co-located on mobile genetic elements (MGEs) alongside antibiotic resistance genes (ARGs) (Baker-Austin et al., 2006; Fard et al., 2011; Holzel et al., 2012; Fang et al., 2016; Song et al., 2017; Argudín et al., 2019), we further investigated the occurrence of genes known to be associated with either antibiotic- or metal resistance on mobile genetic elements among our isolate collection (Supplementary Table S1).

Further investigation included screening of 203 genes described by Pal et al. (2013), particularly known to be associated with increased tolerance or even resistance toward different biocides, for example acridine compound acriflavine [ACR], benzalkonium chloride [BKC] and benzethonium chloride [BEN]. The screening procedure included among others *qac*E, its variant *qac*E(Δ1), *qac*L, *sug*E, *ygi*W, *ymg*B (Supplementary Table S3) and further operons known to be involved in heavy metal detoxification (*ars*ABCD, *cus*ABCF, *mer*RT, *pco*ABCDE, *pco*RS, *sil*ABCEFP, *sil*RS, *ter*BCDWZ, *ygi*W) alongside their regulatory genes (Supplementary Table S3).

Consequently, *in silico* whole-genome screening for all these genes associated with antibiotic resistance (ARGs) [*n* = 2570 included variants of ARGs], biocide resistance or heavy metal tolerance was performed using an in-house BLAST-pipeline with the general identity threshold 95% ID and 90% minimum length based on ResFinder 3.1 (Zankari et al., 2012), CARD (The Comprehensive Antibiotic Resistance Database, Jia et al., 2017) and BacMet (Antibacterial Biocide & Metal Resistance Genes Database; Pal et al., 2013).

Whole genome screening data were used for further genotype characterization including determination of multilocus sequence type (ST) using MLSTFinder 2.0 (Larsen et al., 2012), serotype prediction (SerotypeFinder 2.0, Joensen et al., 2015) and occurrence of plasmid incompatibility groups with PlasmidFinder 2.0 with a threshold of 95% ID (Carattoli et al., 2014). A detailed overview on all isolates and characteristics is provided in Supplementary Table S1.

Whole genome sequences for all 179 *E. coli* are deposited into NCBI-Genbank and accession numbers are provided in Supplementary Table S4.

### Statistical Analysis

Data were analyzed using SPSS software version 25.0 (IBM, New York, NY, United States). *P*-values < 0.05 were considered statistically significant.

A linear mixed-model regression approach was used to test whether feeding group and sampling sites (mucosa, digesta and feces) had an effect on *E. coli* ZnCl_2_ MICs, with the individual pig as a random factor. The logarithm (basis 2) of ZnCl_2_ MIC values was the dependent variable in all analyses. All two-way interactions were included in the models. Non-significant interactions were removed one by one. Variance components were used to determine the proportion of variance that accounted for differences between individual animals. Model diagnostics included visual inspection of residuals for normality and homoscedasticity.

Further, mixed linear regression models were developed to investigate the influence of

• occurrence of resistance toward one or more antibiotic classes (yes or no),
• the MIC levels of acridine compound acriflavine (logarithmic to the basis 2),
• silver nitrate (logarithmic to the basis 2), or
• chlorhexidine (logarithmic to the basis 2), respectively,

on lg2 ZnCl_2_ MIC values including sample origin and feeding group as fixed factors and the individual pig as a random factor.

## Results

### Zinc Tolerance Levels of Porcine Intestinal *E. coli*

**FIGURE 1:**
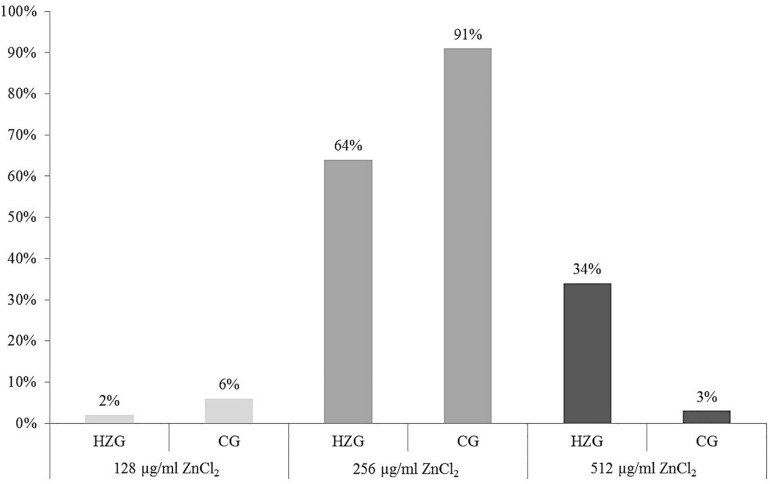
Distribution of zinc chloride MICs among 179 intestinal *E. coli* from piglets. Relative (%) distribution of three different ZnCl_2_ MICs among isolates from the high-zinc group (HZG, *n* = 99) and the control group (CG, *n* = 80).

Here we present results based on a stratified random sample comprising 179 *E. coli* (Supplementary Table S1) representing different AST phenotypes, three different sampling sites (digesta, mucosa, feces) and two feeding groups (HZG and CG). For this collection three different levels of tolerance toward zinc chloride (ZnCl_2_) were detected (Table 1). The lowest level of tolerance toward ZnCl_2_ (128 μg/ml) was recorded for 2% of the HZG and 6% of the CG isolates, respectively. Sixty four percent of the HZG and 91% of the CG isolates were associated with a ZnCl_2_ MIC of 256 μg/ml. Considering the maximum ZnCl_2_ MIC of 512 μg/ml exhibited by isolates in this study, a clear difference between isolates belonging to the distinct feeding groups was obvious: while 34% of the *E. coli* from samples of the HZG clustered here, only 3% of those obtained from the CG reached this particular tolerance level as well (Figure 1).

Further analysis using a linear mixed regression model taking inter-individual host (*n* = 16 pigs) differences (inter-host variance: 16.7% of total variance) into account revealed a statistically significant association between ZnCl_2_ MICs and isolates representing the HZG as well as ZnCl_2_ MICs with isolates of the CG (*p* = 0.005) with a regression coefficient of −0.332 (Table 2). Considering *E. coli* from the three sampling sites (mucosa, digesta from *colon ascendence* and feces from the *ampulla recti*), ZnCl_2_ MICs lacked significant differences (*p* = 0.636).

**TABLE 2:** Results of mixed linear regression model examining the influence of feeding group and sample site on lg2 ZnCl_2_ (dependent factor) for 179 *E. coli* with pig as random variable.

**Factor**	**Regression coefficient**	***p*-value**	**95% CI**
			
Intercept	8.282	<0.001	8.111–8.453
Mucosa	0.006	0.937	−0.137–0.148
Digesta	0.052	0.358	−0.078–0.202
Feces	0^b^	0.636^a^	.
HZG	0^b^	.	.
CG	−0.332	**0.005**	−0.552 – (−0.111)

*HZG, high zinc group; CG, control group; CI, Confidence interval. ^*a*^Global *p*-value determined for three sampling sites (mucosa, digesta, feces). ^*b*^This parameter is set to zero because it is redundant. Bold numbers indicate a significant *p*-value.*

### Zinc Tolerance, Antibiotic and Biocide Susceptibility Profiles

**FIGURE 2:**
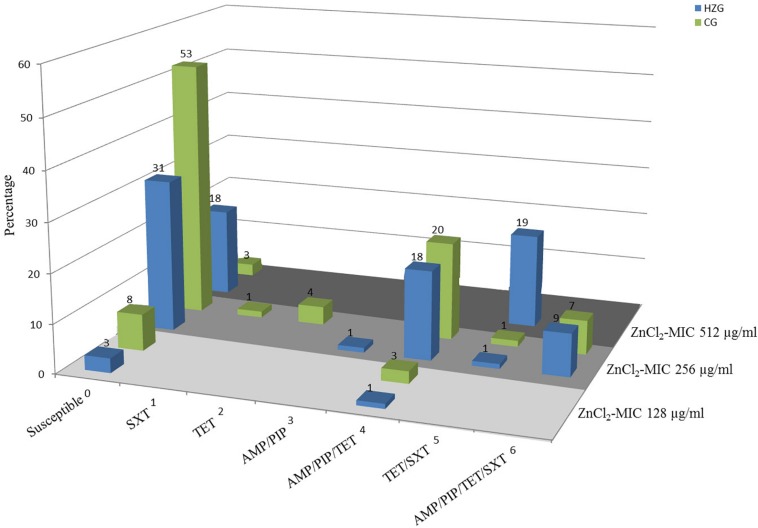
Antibiotic resistance pattern and zinc chloride MICs of porcine intestinal *E. coli.* Relative (%) distribution of resistance pattern compared with zinc chloride MICs (light gray = ZnCl_2_ MIC of 128 μg/ml; gray = ZnCl_2_ MIC of 256 μg/ml; dark gray = ZnCl_2_ MIC of 512 μg/ml) in high-zinc group (blue) [HZG] and control group (green) [CG]. SXT, sulfamethoxazole-trimethoprim; TET, tetracycline; AMP, ampicillin; PIP, piperacillin.

To answer the question whether a particular level of zinc tolerance is associated with a certain antibiotic resistance phenotype, all *E. coli* were grouped according to their individual resistance profile and zinc tolerance level (Figure 2). Non-susceptibilities detected for the 179 *E. coli* included those to sulfamethoxazole-trimethoprim, tetracycline, ampicillin and piperacillin only (Table 1 and Figure 2). Isolates expressing the “highest” ZnCl_2_ MIC (512 μg/ml) showed either a susceptible phenotype toward the panel of antibiotics tested here or yielded non-susceptibility for tetracycline and sulfamethoxazole-trimethoprim. Overall, MICs of antibiotic-resistant (resistance toward one or more antibiotic classes) and susceptible isolates showed no significant difference using mixed linear regression (*p* = 0.119) (Table 3), but drug resistant isolates had slightly higher ZnCl_2_ MIC values than susceptible isolates (regression coefficient 0.105). Overall, zinc tolerance levels of *E. coli* investigated in this study lacked a particular association with the occurrence of any antibiotic resistance phenotype concerning the antimicrobial substances included.

**TABLE 3:** Results of mixed linear regression model examining the influence of antimicrobial resistance, feeding group and sampling site on lg2 ZnCl2 MIC (dependent factor) for 179 *E. coli* with pig as random variable.

**Factor**	**Regression coefficient**	***p*-value**	**95% CI**
			
Intercept	8.343	<0.001	8.156–8.530
Mucosa	0.007	0.926	−0.135–0.149
Digesta	0.064	0.365	−0.075–0.203
Feces	0^b^	0.631^a^	.
HZG	0^b^	.	.
CG	−0.327	**0.006**	−0.548–(−0.106)
Susceptible	−0.105	0.119	−0.236–0.027
Resistant	0^b^	.	.

*HZG, high zinc group; CG, control group; CI, Confidence interval. ^*a*^Global *p*-value determined for the three sampling sites. ^*b*^This parameter is set to zero because it is redundant. Bold numbers indicate a significant *p*-value.*

Minimum inhibitory concentrations detected for biocides and further inorganic metal compounds showed a unimodal value distribution (Table 4), possibly indicating the lack of a distinct non-wild type *E. coli* subpopulation for any of the substances tested here. The broadest distribution of MICs was recorded for acridine compound acriflavine, silver nitrate and chlorhexidine, comprising four dilution steps each (Table 4).

**TABLE 4:** Distribution of biocide- and heavy metal MICs among 179 porcine commensal *E. coli.*

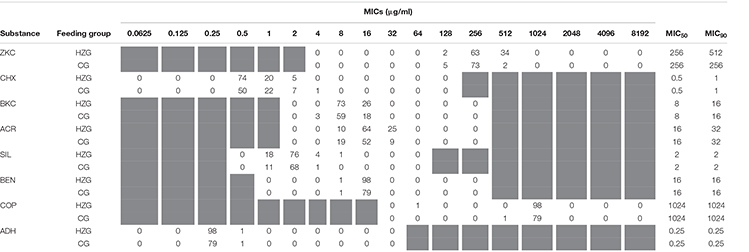

*MIC value ranges for different biocides and heavy metals in μg/ml. Distribution is shown for isolates representing both feeding groups (HZG, high zinc group; CG, control group). ACR, acridine compound acriflavine; ADH, alkyldiaminoethyl glycin hydrochloride; BKC, benzalkonium chloride; BEN, benzethonium chloride; CHX, chlorhexidine; COP, copper sulfate; SIL, silver nitrate; ZKC, zinc chloride. MIC_50_ represents the MIC value at which ≥50% of the isolates in a test population are inhibited, MIC_90_ the MIC value at which ≥90% of the isolates within a test population are inhibited (Turnidge et al., 2006). MICs not included in the range tested are shaded in gray.*

We have set up mixed linear regression models to investigate a putative association between ZnCl_2_ MICs and tolerance levels toward a specific biocide or inorganic metal. The model including lg2 acridine MICs as an influence factor beside the feeding group and the three sampling sites once again showed a significant association between ZnCl_2_ MICs and feeding group (*p* = 0.011), but not with either sampling site (*p* = 0.640) or lg2 acridine MICs (*p* = 0.746) (Supplementary Table 5A).

The silver nitrate model (dependent variable lg2 ZnCl_2_ MIC, independent variables lg2 silver nitrate MIC, feeding group and sampling site) also showed a significant association with the feeding group (*p* = 0.010), but not for lg2 silver nitrate (*p* = 0.979) nor the sampling site (*p* = 0.643, Supplementary Table 5B).

The model build to test the influence of chlorhexidine showed a significant interaction between the feeding group and the lg2 chlorhexidine MICs (*p* = 0.047). While the lg2 ZnCl_2_ MICs decreased with increasing lg2 chlorhexidine MICs in the HZG, they increased slightly in the CG with increasing lg2 chlorhexidine MICs. The parameters sampling site (*p* = 0.338) and feeding group (*p* = 0.137) did not have significant effects, but lg2 chlorhexidine MIC (p = 0.022) did (Supplementary Table 5C). 15.6% of the variance was due to variance between animals.

### Genomic Background and Genes Involved in Zinc Tolerance

Whole genome screening data were used to assign all *E. coli* ST and to predict serotypes among the isolates representing the HZG (*n* = 99) and the CG (*n* = 80). As shown in Figure 2, these 179 isolates representing the two feeding groups were assigned to 15 STs each. Overall, isolates belonging to the sequence type complex (STC) 10 (ST10 and ST34) were most common among the representative isolates of both groups (HZG, 50.5%; CG, 40%).

**FIGURE 3:**
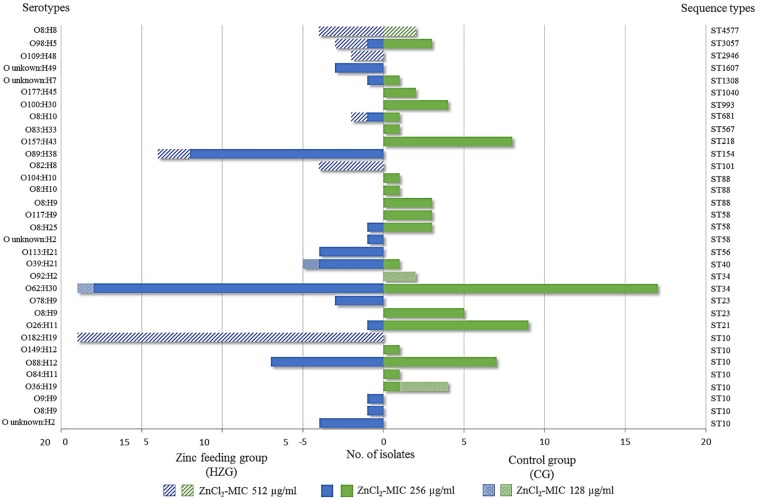
Sequence types and serotypes of 179 with *E. coli* showing three different zinc chloride MICs. Sequence- and (predicted) serotype distribution and frequencies of 179 *E. coli* isolates representing both feeding groups together with zinc chloride tolerance MICs. Most isolates showed the ZnCl_2_ MIC of 256 μg/ml, represented by 17 STs in both feeding groups. The broadest heterogeneity seems to be associated with sequence type complex (STC)10 isolates, which were obtained from both feeding groups (high-zinc group [HZG] and control group [CG]), including representatives for each of the three zinc chloride tolerance values.

Considering the corresponding predicted serotypes, *E. coli* O62:H30 (ST34) was common in the representative samples of both feeding groups (20% [HZG]/19% [CG]), followed by the ST10 O88:H12 (9% [HZG]/7% [CG]) (Figure 2). In addition, most *E. coli* representing both feeding groups (141 of 179) showed a ZnCl_2_ MIC of 256 μg/ml. These isolates were assigned to 17 STs and 27 serotypes. The isolates with ZnCl_2_ MIC 128 μg/ml belonged to three different STs (4 serotypes) and the ZnCl_2_ MIC of 512 μg/ml included isolates of seven STs and seven corresponding serotypes (Figure 3). Both groups of *E. coli* representing the two different feeding groups included predicted serotypes which were unique to it, for instance CG isolates belonging to O92:H2 and O157:H43 or O89:H38 and O182:H19 in the HZG (Figure 3).

In particular factors have been described as being capable to confer elevated zinc tolerance levels in *E. coli* (Grass et al., 2005; Deus et al., 2017; Vidhyaparkavi et al., 2017; Stocks et al., 2019). Consequently, we analyzed the presence or absence of a broad set of genes involved in zinc homeostasis (*n* = 35) (Table 5) including zinc-binding metalloenzymes (*n* = 69) (Supplementary Table S2). All 179 isolates harbored genes associated with zinc detoxification such as *zit*B, *znt*A and *pit* (Table 5). Also, factors involved in zinc uptake like ZupT (metal uptake protein, preference for zinc), the ABC transporter ZnuABC and major regulators such as Zur were identified in each of the genomes. Only the Rac-prophage zinc-binding chaperone protein YdaE (Blindauer et al., 2002) and the Zn(II)-responsive ribosomal proteins YkgM (Hensley et al., 2012) was not ubiquitously distributed among isolates of the three distinct ZnCl_2_ MICs (Table 5). In addition, almost all genomes were found positive for genes encoding zinc-binding metalloenzymes involved in a highly diverse net of cell functions (Supplementary Table S2). A detailed overview on gene presence or absence, query protein coverage and number of predicted protein variants for these 104 factors investigated here is provided in Table 5 and Supplementary Table S2. Accordingly, sheer presence or absence of particular factors or even a particular amino acid sequence variant does not explain the different levels of zinc tolerance among the analyzed *E. coli* population.

**TABLE 5:** Screening results of factors involved in zinc homeostasis for 179 porcine commensal *E. coli.*

			**Distribution**			**Protein**				
					
			**ZnCl_2_-MICs (μg/ml)**							
			
			**128**	**256**	**512**					
**Function**	**Symbol**	**Gene**	***n* = 7^*a*^**	***n* = 136^*a*^**	***n* = 36^*a*^**	**L**	**AV**	**C**	**I**	**REF**
			**%**	**%**	**%**	**AA**	***n***	**%**	**%**	
**Zinc uptake**										
Metal binding protein	ZinT	*zin*T	100	100	100	216	14	100	98.4	[1]
Zn-binding protein (ABC)	ZnuA	*znu*A	100	100	100	311	11	100	98.4	[2]
Integral subunit (ABC)	ZnuB	*znu*B	100	100	100	252	11	100	98.5	[2]
ATPase subunit (ABC)	ZnuC	*znu*C	100	100	100	261	5	100	99.0	[2]
Zn^2+^ uptake transporter	ZupT	*zup*T	100	100	100	257	2	100	99.6	[3]
Zn^2+^ uptake regulator	Zur	*zur*	100	100	100	172	10	100	96.4	[4]
Ammonia channel	AmtB	*amt*B	100	100	100	428	5	100	99.9	[5]
Put. arylsulfatase	AslA	*asl*A	100	100	100	552	11	100	98.5	[6]
OM channel	OmpC	*omp*C	100	100	100	368	10	100	90.8	[5]
Put. protein	YdfE	*ydf*E	100	100	100	255	19	100	96.1	[6]
Efflux protein (cysteine)	EamB	*eamB*	100	100	100	195	10	100	98.7	[5]
**Efflux**										
AMG efflux pump	AcrD	*acr*D	100	100	100	1037	15	100	99.8	[7]
MDR transporter	MdtA	*mdt*A	100	100	100	415	20	100	99.2	[7]
MDR transporter	MdtB	*mdt*B	100	100	100	1040	24	100	98.1	[7]
MDR transporter	MdtC	*mdt*C	100	100	100	1025	24	100	98.0	[7]
MDR transporter	MdtD	*mdt*D	100	100	100	471	20	100	98.4	[7]
Ferrous-iron efflux pump	FieF	*yiip*	100	100	100	300	4	100	99.7	[8]
Metal transporter	ZitB	*zit*B	100	100	100	314	10	100	99.6	[9]
P1b-type ATPase	ZntA	*znt*A	100	100	100	732	13	100	97.4	[7]
Histidine-protein kinase	BaeS	*bae*S	100	100	100	467	10	100	94.3	[7]
Transcriptional regulator	BaeR	*bae*R	100	100	100	240	10	100	99.3	[7]
Transcriptional regulator	SoxS	*sox*S	100	100	100	108	8	100	99.5	[10]
Transcriptional activator	SoxR	*sox*R	100	100	100	154	8	100	99.5	[10]
Transcriptional regulator	ZntR	*znt*R	100	100	100	142	12	100	98.9	[11]
Transglycosylase E	EmtA	*emt*A	100	100	100	203	4	100	99.7	[6]
Formate dehydrogenase	FdnG	*fdn*G	100	100	100	1016	9	100	99.5	[6]
Put. Zn^2+^ protease	PqqL	*pqq*L	100	100	100	932	10	100	98.1	[12]
GTP cyclohydrolase II	RibA	*rib*A	100	100	100	197	7	100	99.8	[6]
Periplasmic chaperone	Spy	spy	100	100	100	159	9	100	99.7	[5]
Put. Zn^2+^ chaperone	YdaE	*yda*E	42.9	16.2	52.8	57	3	100	85.0	[5]
Zn^2+^-stimulated GTPase	YeiR	*yei*R	100	100	100	328	6	100	99.4	[13]
50S ribosomal protein	YkgM	*ykg*M	57.1	99.3	100	88	8	100	98.4	[14]
Zn^2+^ resistance as. protein	ZraP	*zra*P	100	100	100	142	11	100	98.0	[5]
Transcriptional regulator	ZraR	*zra*R	100	100	100	441	17	100	99.2	[15]
Sensor protein	ZraS	*zra*S	100	100	100	441	15	100	98.5	[15]

*All 179 porcine commensal *E. coli* were screened with respect to the presence or absence of 35 factors involved in zinc homeostasis as described before. Predicted amino acid sequence lengths were compared to those of *E. coli* K-12 MG1655 in order to check for putative premature stop codons or deletions affecting the putative function of the protein. None of the amino acid sequence variations (AV) was solely associated with a particular ZnCl_2_ MIC. Amino acid sequence variation among the isolate collection primarily reflects the isolate’s genetic backgrounds. Put., putative; OM, outer membrane; MDR, multidrug resistance; AMG, aminoglycoside; as., associated; n, number of isolates; ^*a*^, number of isolates with particular ZnCl_2_ MIC; L, length; AA, amino acid sequence; AV, number of amino acid sequence variants; C, amino acid sequence coverage with respect to reference protein in *E. coli* K-12 MG1655; I, maximum amino acid sequence identity among the 179 isolates; REF, references. [1] Colaço et al., 2016; [2] Yatsunyk et al., 2008; [3] Grass et al., 2005; [4] Choi et al., 2017; [5] Lee et al., 2005; [6] Graham et al., 2009; [7] Wang and Fierke, 2013; [8] Lu et al., 2009; [9] Watly et al., 2016; [10] Warner and Levy, 2012; [11] Wang et al., 2012; [12] Subashchandrabose et al., 2013; [13] Blaby-Haas et al., 2012; [14] Hensley et al., 2012; [15] Petit-Hartlein et al., 2015.*

### Antibiotic Resistance – Biocide – And Heavy Metal Tolerance Genes

In total, 87/179 *E. coli* comprising isolates of both feeding groups and each zinc tolerance level lack ARGs, which is in strict concordance with their resistance phenotype (Figure 2 and Table 6). However, genes known to confer antibiotic resistance have been identified on MGEs within the isolate collection. While the ZnCl_2_ MIC 128 μg/ml is associated with the occurrence of 0–4 ARGs and three STs, the ZnCl_2_ MIC 256 μg/ml shows a range from 0 to7 ARGs and ZnCl_2_ MIC 512 μg/ml harbors either 0 or 5 ARGs (Supplementary Table S1).

**TABLE 6:** Antibiotic resistance genes (ARGs).

			**Distribution**			**Protein**				
					
			**ZnCl_2_-MICs (μg/ml)**							**REF**
								
**Factor**	**Symbol**	**Gene**	**128**	**256**	**512**					
			***n* = 7^*a*^**	***n* = 136^*a*^**	***n* = 36^*a*^**	**L**	**AV**	**C**	**I**	
			**%**	**%**	**%**	**AA**	**n**	**%**	**%**	
Streptomycin 3′′-adenyltransferase	AadA1	*aad*A1	28.6	18.4	52.8	263	2	100	99.2	[1]
Aph(3′′)-Ib PT	APH(3′′)-Ib	*str*A	14.3	38.2	0	267	2	100	99.9	[2]
Aph(6)-Id PT	APH(6)-Id	*str*B	14.3	38.2	0	287	1	100	100	[2]
Tetracycline resistance protein	Tet(A)	*tet*(A)	0	11.0	52.8	399	2	100	99.9	[3]
Tetracycline resistance protein	Tet(B)	*tet*(B)	14.3	29.4	0	401	1	100	99.9	[4]
Macrolide 2′-PT II	MPH(2‘)-II	*mph*(B)	0	2.2	0	302	1	100	100	[5]
β-lactamases	BlaTEM-1b	*bla*TEM-1b	0	30.9	0	286	1	100	98.6	[6]
Dehydrofolate reductase	DfrA1	*dfr*A1	0	11.8	52.8	157	2	100	99.9	[7]
Dihydropteroate synthase-type 1	DHPS-1	*sul*1	0	8.1	52.8	279	2	100	99.9	[8]
Dihydropteroate synthase-type 2	DHPS-2	*sul*2	0	0.7	0	271	1	100	99.4	[8]
Dihydropteroate synthase-type 3	DHPS-3	*sul*3	0	9.6	52.8	263	1	100	99.9	[8]

**FIGURE 4:**
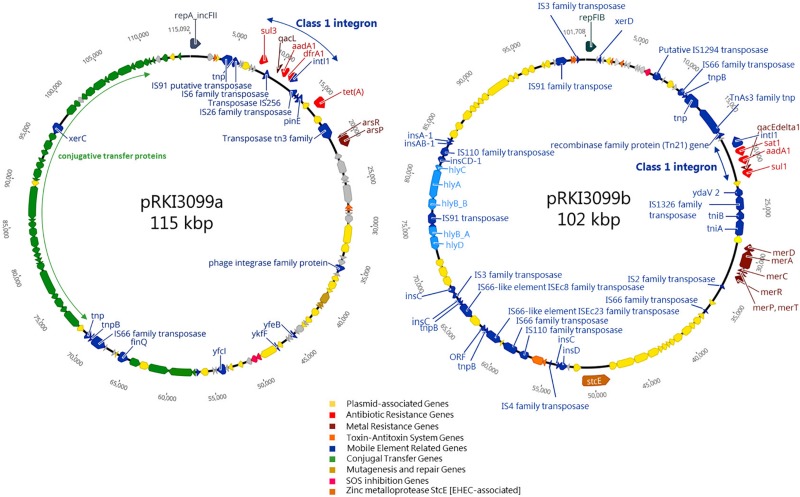
Schematic circular representation of plasmids pRKI3099a and pRKI3099b from *E. coli* isolate RKI3099. RKI3099 belonged to ST10 O182:H19 expressing ZnCl_2_ MIC of 512 μg/ml. The predicted protein function are indicated by color code as follows: plasmid-association (yellow), antibiotic resistance (red), metal resistance (dark red), toxin-antitoxin system (orange), mobile genetic elements (blue), conjugal transfer (green), mutagenesis and repair (gold), SOS inhibition (pink) and the zinc metalloprotease StcE (brown). Plasmid pRKI3099a (115 kpb; IncFII) and pRKI3099b (102 kbp; IncFIB) carry class 1 integron variants by integrase IntI1 (small blue arrows), and a disrupted form of the biocide resistance gene *qac*E (*qac*EΔ1) or the biocide resistance gene *qac*L, as well as the sulfonamide resistance genes *sul*1 or *sul*3.

*Whole genome sequencing data of 179 porcine commensal *E. coli* were screened with respect to antibiotic resistance genes (ARGs). Predicted amino acid sequence lengths were compared to those of the ResFinder 3.1 data base (Zankari et al., 2012) in order to check for putative premature stop codons or deletions affecting the putative function of the protein. None of the amino acid sequence variations (AV) was solely associated with a particular ZnCl_2_ MIC. Isolates with the 512 μg/ml ZnCl_2_ MIC which were positive for plasmid-associated ARGs exclusively belonged to ST10 O182:H19 (Figure 4). n, number of isolates; ^*a*^, number of isolates with particular ZnCl_2_ MIC; L, length; AA, amino acid; AV, number of amino acid sequence variants; C, amino acid sequence coverage with respect to ResFinder 3.1 data base (Zankari et al., 2012); I, maximum amino acid sequence identity among the 179 isolates; REF, references. [1] Hollingshead and Vapnek, 1985; [2] Scholz et al., 1989; [3] Aldema et al., 1996; [4] Roberts, 2005; [5] Pawlowski et al., 2018; [6] Salverda et al., 2010; [7] Sköld, 2001; [8] Antunes et al., 2005.*

Seven isolates expressing a ZnCl_2_ MIC of 128 μg/ml belonged to ST10 O36:H19 (*n* = 3), ST34 O62:H30 (*n* = 1), O92:H2 (*n* = 2), and ST40 O39:H21 (*n* = 1). ARGs conferring resistance to aminoglycosides, beta-lactams and tetracycline (*aad*A1, *bla*_*TEM–*__1__*b*_, *str*A, *str*B, and *tet*B) were solely associated with both serotypes belonging to ST34.

Sixty nine of 136 *E. coli* with the 256 μg/ml ZnCl_2_ MIC harbored a different ARG combinations, e.g., isolates belonging to ST56 O113:H21 (*n* = 4) were positive for *aad*A1 and *sul*1 and ST993 O100:H30 (*n* = 4) isolates harbored *tet*B. ST10 O88:H12 (*n* = 13) harbored *aad*A1, *dfr*A1, *bla*_*TEM–*__1__*b*_, *str*A, *str*B, *sul*3, and *tet*A.

Considering the 36 isolates with the 512 μg/ml ZnCl_2_ MIC, ARGs (*aad*A1, *dfr*A1, *sul*1, *sul*3 and *tet*A) conferring resistance to aminoglycosides, trimethoprim, sulfonamides and tetracycline were exclusively associated with 19 isolates belonging to ST10 O182:H19. The remaining 17 isolates completely lack ARGs and belong to six different STs (101, 154, 681, 2946, 3057, 4577) (Supplementary Table S1). WGSc indicated the presence of more than one plasmid harboring the 5 ARGs and further genes conferring tolerance toward biocides and heavy metals (Figure 4) among the ST10 O182:H19 isolates. Subsequently, PacBio sequencing was employed to further investigate the composition of these structures extracted from a representative isolate (RKI3099).

As a result, two novel plasmids (Figure 4) with a size of 115 kb (pRKI3099a; accession number MN124285) and 102 kb (pRKI3099b; accession number MN124286) were identified. Both plasmids carry class I integron variants characterized by integrase IntI1, and either a disrupted form of the biocide resistance gene *qac*E (*qac*EΔ1) (Paulsen et al., 1996) or the biocide resistance gene *qac*L, as well as the sulfonamide resistance genes *sul*1 (RKI3099b) or *sul*3 (pRKI3099a). The plasmid pRKI3099a is a IncFII-type plasmid that also carries the tetracycline resistance gene *tet*A and the nearly 40 kb transfer operon (*tra*) which is essential for F-plasmid transfer (Frost et al., 1994). The IncFIB plasmid RKI3099b harbors the mercury resistance operon *mer*RTPCDAB, diverse insertion sequences and a *hly-operon* disrupted by IS91. Additionally, both plasmids include typical plasmid partitioning genes such as toxin-antitoxin systems (e.g., *vap*B/C and *hic*A/B) and further mobile element related genes (Figure 4).

Taken together, the occurrence of certain ARGs is more associated with the genomic background and serotype of the *E. coli* investigated here than with a particular zinc tolerance level or feeding group. Consequently, we decided to abstain from further statistic calculation for individual resistance genes clearly mirroring frequencies of certain genomic lineages only.

Genes known to confer resistance/tolerance toward biocides and heavy metals located on plasmids seem to have a strong association (*p* ≤ 0.05) with isolates of the HZG, comprising different STs and serotypes (Supplementary Table S1). However, considering the ZnCl_2_ MIC of 512 μg/ml within the HZG, only isolates belonging to ST10 O182:H19 carried the above mentioned plasmids harboring biocide resistance (e.g., quaternary ammonium compound efflux transporters encoded by *qac*L or *qac*EΔ1) and heavy metal tolerance genes (e.g., mercury resistance operon *mer*RTPCDAB) alongside further ARGs (Figure 4).

## Discussion

Former studies revealed an increase in antibiotic resistance among *E. coli* (Holzel et al., 2012; Bednorz et al., 2013) and other bacteria (Aarestrup et al., 2010; Cavaco et al., 2010) obtained from pig manure which were previously exposed to high zinc supplemented diets. Although a general correlation between heavy metal – and antibiotic resistance seems to exist (Pal et al., 2015; Yu et al., 2017), so far only a few factors conferring heavy metal resistance in different bacterial species have been identified, mostly located alongside ARGs on mobile genetic elements such as insertion sequences and composite transposons (Rice and Carias, 1998), unit transposons (Rubens et al., 1979; Liebert et al., 1999), gene cassettes and integrons (Kholodii et al., 2003; Petrova et al., 2011) and plasmids (Carattoli, 2013; Fernandez-Lopez et al., 2016). A prominent example might be the zinc-resistance factor Crz identified on the staphylococcal chromosomal cassette conferring methicillin resistance (SCC*mec* type V) in *S. aureus* from livestock (Cavaco et al., 2011).

### Zinc Tolerance Levels, Antibiotic- and Biocide Susceptibility of Porcine Intestinal *E. coli*

Here we report about phenotypic and genotypic characteristics of 179 *E. coli* selected from a collection obtained from piglets fed with either a zinc-rich diet (*n* = 8) or a common piglet diet (*n* = 8). Considering the ZnCl_2_ MICs in this study, our isolate collection seems to reflect the naturally occurring unimodal distribution of zinc tolerance levels most likely lacking non-wildtype phenotypes, as previously reported for *E. coli* of avian and human origin (Deus et al., 2017). So far, an association between a ZnCl_2_ MIC and a particular antibiotic resistance phenotype has not been detected.

Nonetheless, the linear mixed regression model revealed a statistically significant association between “higher” ZnCl_2_ MICs and isolates representing the HZG as well as “lower ZnCl_2_ MICs” with isolates of the CG (*p* = 0.005), indicating a selective advantage of distinct commensal *E. coli* lineages in the presence of high amounts of zinc in the piglets’ diet. One exception is *E. coli* belonging to ST4577 (O8:H8). This genotype shows a 512 μg/ml zinc MIC, and while it occurs in samples from both feeding groups, it is nonetheless only rarely found among CG isolates (2.5%) (Figure 3).

A recent study showed that zinc inhibits virulence expression of diarrheagenic *E. coli* by inducing the bacterial envelope stress response while inhibiting the SOS response (Bunnell et al., 2017). Moreover, zinc salts were found to be capable of reducing SOS-induced hypermutation through error-prone polymerases in the presence of antibiotics (Bunnell et al., 2017). Consequently, isolates exhibiting comparatively higher levels of zinc tolerance might reflect a more inert SOS-system activation, gaining a selective advantage by preventing early SOS-induced hypermutation, even at sub-inhibitory concentrations of antibiotics. Clearly, further studies on that particular subject are needed, as our comprehensive *in silico* analysis did not give any explanation about which mechanism might be responsible for the variety of zinc tolerance measured.

The distribution of MICs obtained for further heavy metals and different biocides suggested the lack of a non-wild type population among the isolate collection, as assumed before (Deus et al., 2017). The mixed linear regression model set-up for lg2 chlorhexidine MICs indicated a putative association of lg2 chlorhexidine MICs and lg2 ZnCl_2_ MICs in the different feeding groups. However, the complex nature of interactions between biocides and heavy metals and their possible effects on bacterial populations needs to be further investigated.

### Genomic Background and Genes Involved in Zinc (Heavy Metal)- and Biocide Tolerance

*Escherichia coli* belonging to the STC10 (ST10 and ST34) were most common among the isolates of both groups (HZG, 50.5%; CG, 40%). This finding is in accordance with a recent study which summarizes that in Germany, Denmark, Ireland, and Spain STC10 is the dominant genomic lineage among commensal *E. coli* from pigs (Cortés et al., 2010; Bednorz et al., 2013; Herrero-Fresno et al., 2015; Wang et al., 2016; Ahmed et al., 2017) and reported this lineage as being predominant among intestinal *E. coli* from Australian pigs’ as well (Reid et al., 2017). Moreover, *E. coli* belonging to STC10 have been characterized as opportunistic, frequently associated with multidrug resistance and widely distributed among a broad host range (Alcalá et al., 2016; Cordoni et al., 2016; Guenther et al., 2017).

Based on former studies, several genes have been emphasized with respect to their capability to confer increased zinc tolerance or even -resistance in *E. coli*. In this context, the P1b-type ATPase ZntA and the CDF ZitB have been frequently nominated (Hantke, 2005; Ding et al., 2012; Porcheron et al., 2013; Watly et al., 2016; Deus et al., 2017; Ojer-Usoz et al., 2017). In addition, the protein ZraP associated with zinc-resistance, the transcriptional regulatory protein ZraR (synonym: HydG), the serine acetyltransferase CysE and the low-affinity inorganic phosphate transporter PitA have been linked to zinc tolerance (Casewell et al., 2003; Hoegler and Hecht, 2018; Stocks et al., 2019). We were able to provide evidence that genes encoding these and many other factors involved in bacterial zinc hemostasis were present in almost all 179 isolates investigated, indicating that the sheer presence of these factors or even certain combinations do not confer zinc tolerance MICs deviating from the unimodal distribution.

As was described for ZupT, point mutations are able to change the kinetics of metal uptake systems toward an increase zinc tolerance (Taudte and Grass, 2010). However, isolates investigated here lack any ZupT amino acid sequence variation (Table 5). Considering the predicted amino acid variations of other zinc-associated proteins, differences seem to be lineage-specific and lack particular associations with the distinct zinc MICs (Table 5).

Accumulations of metals such as copper and zinc have also been linked to antibiotic resistance development in environmental bacteria, as thoroughly reviewed by Poole (2017). However, molecular mechanisms responsible for the presence as well as absence of heavy metal-, antibiotic-, and biocide co-resistances are not fully understood yet (Yu et al., 2017). A putative effect of overexpressing non-specific efflux pumps conferring phenotypic resistance to antibiotics, biocides and heavy metals, as described for *Listeria monocytogenes* (Mata et al., 2000) to the phenotypes reported here cannot be ruled out completely. Therefore, studying the transcriptomic response of distinct genomic lineages with respect to different ZnCl_2_ MICs might reveal the most relevant factors explaining our results. However, for each of the antibiotic resistance phenotypes detected in our isolates at least one corresponding ARG was identified (Supplementary Table S1), clearly reasoning against a primary role of any non-specific efflux pumps.

In the past, there has been an extensive and substantial discussion about fitness cost(s) of ARGs for bacteria, especially *E. coli* (Melnyk et al., 2015), which is beyond the scope of this project. However, the ZnCl_2_ MIC 512 μg/ml value is associated with either ST10 O182:H19 and the ARGs *aad*A1, *dfr*A1, *sul*1, *sul*3, and *tet*A (Figure 4) conferring resistance to aminoglycosides, trimethoprim, sulfonamides and tetracycline or no ARG at all (6 STs; 6 serotypes) (Supplementary Table S1 and Figure 2). Notably, most of these ARGs in ST10 O182:H19 are associated with class I integrons, which are commonly regarded as “low-cost structures,” known to promote selection while confronted with sub-inhibitory antibiotic concentrations (Lacotte et al., 2017). Thus, picturing a co-selective pressure above the minimal selective pressure induced by zinc excess together with any of the antibiotics mentioned, a clear selective advantage, even if associated with cost(s), seems reasonable for this serotype, as it was discussed for appearance and elimination of resistance genes before (Yu et al., 2017). Moreover, a former study from Australia revealed commensal *E. coli* from pigs as a reservoir for class 1 integrons, frequently associated with three or more ARGs as well as genes conferring heavy metal tolerance (Reid et al., 2017). As proposed, biocides may promote dissemination of mobile genetic elements and hence resistance genes (Gillings et al., 2008). Moreover, biocides may have driven the fixation and spread of class 1 integrons, responsible for a major part of antibiotic resistance (Gillings et al., 2008).

Almost all 179 *E. coli* described here harbored the chromosomally encoded AcrAB-TolC-system which is known to decrease susceptibility toward a wide variety of antibiotics and biocides including acriflavine (Buffet-Bataillon et al., 2012). While detoxification by overexpression of AcrAB-TolC and other efflux pumps (e.g., MdtEF-TolC) has been reported before (Novoa and Conroy-Ben, 2019), this study aimed to evaluate the genes whose presence were described as responsible or involved in increasing bacterial zinc tolerance (Table 5), an assumption we have clearly rejected. Nonetheless, regulatory proteins such as SoxS for AcrAB are sensitive for oxidative stress (Harrison et al., 2009) induced by different metal ions, subsequently leading to an increased expression of the corresponding efflux system while mediating tolerance toward a broad range of antibiotics (Seiler and Berendonk, 2012). Consequently, further research on differences in transcription patterns during zinc-induced stress might reveal the factors essential for increased zinc tolerance in particular *E. coli* lineages.

## Conclusion

Using comprehensive phenotypical and *in silico* analyses, this study sheds light on the effects of high-zinc oxide diets on intestinal *E. coli* populations in weaned piglets: An association of the isolates’ ZnCl_2_ MIC with the feeding group was obvious, while neither the presence nor the rare absence of a specific gene or gene combination involved in cellular zinc homeostasis could be identified to be associated with a particular degree of zinc tolerance. Thus, a simple model of co-selection does not account for the different levels of zinc tolerance reported here.

## Data Availability Statement

The datasets generated for this study can be found in the NCBI database. A full list for all 179 entries is provided in Supplementary Table S4.

## Author Contributions

LW, AL-B, and AB designed the project. VJ and BW conceived and designed the experiments. IE sequenced the isolates. VJ performed the laboratory analysis. VJ, BW, LE, TS, FG, RM, and YP analyzed the data. VJ, BW, and LW wrote the manuscript. All authors have read and approved the final draft of the manuscript.

## Conflict of Interest

The authors declare that the research was conducted in the absence of any commercial or financial relationships that could be construed as a potential conflict of interest.
