# Supplementary material for: Effects of a Four-Week High-Dosage Zinc Oxide Supplemented Diet on Commensal Escherichia coli of Weaned Pigs
Source: Front Microbiol. 2019 Nov 28;10:2734. doi: 10.3389/fmicb.2019.02734 (PMC6892955; doi:10.3389/fmicb.2019.02734)
Supplement: Supplementary file 2 [file Table_2.docx]

| **Supplementary Table S2: Zinc-binding metalloenzymes** | | | | | | | | | | | | |
| --- | --- | --- | --- | --- | --- | --- | --- | --- | --- | --- | --- | --- |
|  |  |  |  | **Gene** | | | | **Protein** | | | |  |
|  |  |  |  | **ZnCl_2_-MICs (µg/ml)** | | | |  | | | |  |
|  |  |  |  | **128** | **256** | | **512** |  |  |  |  |  |
| **Function** |  | **Symbol** | **Gene** | **n=7** | **n=136** | | **n=36** | **L** | **AV** | **C** | **I** | **REF** |
|  |  |  |  | **%** | **%** | | **%** | **AA** | **n** | **%** | **%** |  |
| Acetyl-CoA biosynthetic process | Acetate kinase | AckA | *ack*A | 100 | 100 | 100 | | 400 | 4 | 100 | 99.7 | [1] |
| Fatty acid biosynthesis process | Acyl carrier protein phosphodiesterase | AcpH | *acp*H | 100 | 100 | 100 | | 193 | 7 | 100 | 98.8 | [2] |
| DNA repair | Bifunctional transcriptional activator | Ada | *ada* | 100 | 100 | 100 | | 354 | 15 | 100 | 98.7 | [3] |
| Ethanol oxidation, formaldehyde catabolic process | S-(hydroxymethyl)glutathione dehydrogenase | FrmA | *adh*C (*frm*A) | 100 | 100 | 100 | | 369 | 5 | 100 | 98.8 | [4] |
| Alanyl-tRNA aminoacyla-tion (interpreta-tion of genetic code) | Alanyl-tRNA synthetase | AlaS | *ala*S | 100 | 100 | 100 | | 876 | 10 | 100 | 99.2 | [5] |
| Nitrogen metabolism | Allantoate amidohydrolase | AllC | *all*C | 57.1 | 96.3 | 100 | | 411 | 15 | 100 | 98.5 | [6] |
| Cell wall organisation | anhydro-N-acetylmuramoyl-l-alanine amidase | AmiD | *ami*D | 100 | 100 | 100 | | 276 | 8 | 100 | 99.2 | [7] |
| Cell wall organisation | N-acetyl-anhydromuramyl-L-alanine amidase | AmpD | *amp*D | 100 | 100 | 100 | | 183 | 8 | 100 | 99.7 | [8] |
| L-arginine biosynthesis | acetylornithine deacetylase | ArgE | *arg*E | 100 | 100 | 100 | | 383 | 7 | 100 | 99.1 | [9] |
| Carbon utilization | carbonic anhydrase | Can | *can* | 100 | 100 | 100 | | 220 | 1 | 100 | 100 | [10] |
| Cytidine deamination | Cytidine deaminase | Cdd | *cdd* | 100 | 100 | 100 | | 294 | 5 | 100 | 99.8 | [11] |
| Carbon utilization | Carbonic anhydrase | CynT | *cyn*T | 100 | 96.3 | 97.2 | | 219 | 6 | 100 | 99.7 | [12] |
| Cysteinyl-tRNA aminoacyla-tion (interpretion of genetic code) | Cysteinyl-tRNA synthetase | CysS | *cys*S | 100 | 100 | 100 | | 461 | 6 | 100 | 99.8 | [13] |
| L-lysine biosynthesis via DAP pathway (bacterial cell wall) | N-Succinyl-L-diaminopimelate desuccinylase | DapE | *dap*E | 100 | 100 | 100 | | 375 | 10 | 100 | 99.9 | [14] |
| DNA replication (synthesis of RNA primer) | ssDNA-dependent RNA-polymerase | DnaG | *dna*G | 100 | 100 | 100 | | 581 | 6 | 100 | 99.9 | [15] |
| Repair, DNA replication | Chaperone Hsp70 (heat shock 70 kDa) | Dank | *dna*K | 100 | 100 | 100 | | 638 | 4 | 100 | 99.5 | [1] |
| dUMP biosynthesis | Deoxyuridine triphosphatase (dUTPase) | Dut | *dut* | 100 | 100 | 100 | | 151 | 2 | 100 | 99.8 | [16] |
| rRNA processing | Zinc phosphodiesterase | Rbn | *ela*C (*rbn*) | 100 | 100 | 100 | | 305 | 8 | 100 | 99.2 | [17] |
| Gluconeogenesis and glycolytic process | Fructose-bisposphate aldolase, class II | FbaA | *fba*A | 100 | 100 | 100 | | 359 | 2 | 100 | 99.98 | [18] |
| One-carbon metabolic process | GTP cyclohydrolase | FolE | *fol*E | 100 | 100 | 100 | | 222 | 2 | 100 | 99.98 | [19] |
| D-arabinose and L-fucose catabolic process | L-fuculose-phosphate aldolase & D-ribulose-phosphate aldolase | FucA | *fuc*A | 100 | 100 | 100 | | 215 | 4 | 100 | 99.4 | [20] |
| Galactose metabolism | Galactose-1-phosphate uridylyltransferase | GalT | *gal*T | 100 | 100 | 100 | | 348 | 13 | 100 | 98.8 | [21] |
| Methylgly-oxal catabolic process to D-lactate via S-lactoyl-glutathione | Hydroxyacylglutathione hydrolase | GloB | *glo*B | 100 | 100 | 100 | | 251 | 11 | 100 | 99.1 | [22] |
| Glutamyl-tRNA aminoacyla-tion | Glutamyl-tRNA-synthetase | GltX | *glt*X | 100 | 100 | 100 | | 471 | 4 | 100 | 99.4 | [23] |
| Glycine biosynthetic process from serine | Serine hydroxmethyltransferase (SHMT) | GlyA | *gly*A | 100 | 100 | 100 | | 417 | 1 | 100 | 100 | [1] |
| Guanine catabolic process | Guanine deaminase | GuaD | *gua*D | 100 | 99.3 | 100 | | 439 | 8 | 100 | 99.4 | [24] |
| Heme biosynthetic process | Porphobilinogen synthase | HemB | *hem*B | 100 | 100 | 100 | | 324 | 2 | 100 | 99.96 | [25] |
| Proteolysis | Integral membrane ATP-dependent zinc metallopeptidase | FtsH | *hfl*B (*fts*H) | 100 | 100 | 100 | | 644 | 1 | 100 | 100 | [26] |
| Cellular protein modification process | Hydrogenase maturation regulator | HypA | *hyp*A | 100 | 100 | 100 | | 116 | 3 | 100 | 99.9 | [27] |
| Cellular protein modification process | **I**so**a**spartyl **d**ipeptidase | IadA | *iad*A | 100 | 100 | 100 | | 390 | 18 | 100 | 99.4 | [28] |
| Cellular response to DNA damage stimulus | Isopentenyl diphosphate isomerase | Idi | *idi* | 100 | 100 | 100 | | 182 | 10 | 100 | 97.9 | [29] |
| Isoleucyl-tRNA aminoacyla-tion | Isoleucyl-tRNA synthetase | IleS | *ile*S | 100 | 100 | 100 | | 938 | 14 | 100 | 99.0 | [30] |
| Lipid A biosynthesis | UDP-3-O-acyl-N-acetylglucosamine deacetylase | LpxC | *lpx*C | 100 | 100 | 100 | | 305 | 2 | 100 | 99.99 | [31] |
| Lysyl-tRNA aminoacyla-tion | Lysine-tRNA ligase | LysU | *lys*U | 100 | 100 | 100 | | 505 | 7 | 100 | 99.2 | [32] |
| cell wall manno-protein biosynthetic process | mannose-6-phophate isomerase | ManA | *man*A | 100 | 100 | 100 | | 391 | 10 | 100 | 99.0 | [33] |
| Methionine biosynthetic process | homocysteine transmethylase | MetE | *met*E | 100 | 100 | 100 | | 753 | 11 | 100 | 99.4 | [34] |
| Methionyl-tRNA aminoacyla-tion | Methionyl-tRNA synthetase | MetG | *met*G | 100 | 100 | 100 | | 677 | 11 | 100 | 99.7 | [35] |
| DNA repair (base-excision repair) | Formamidopyrimidine DNA glycosylase | MutM | *mut*M | 100 | 100 | 100 | | 269 | 5 | 100 | 99.6 | [36] |
| Carbohydrate metabolic process | **N**-**A**cetyl**g**lucosamine-6-phosphate deacetylase | NagA | *nag*A | 100 | 100 | 100 | | 382 | 10 | 100 | 99.2 | [37] |
| DNA repair (base-excision repair) | Endonuclease VIII | Nei | *nei* | 100 | 100 | 100 | | 263 | 12 | 100 | 98.3 | [38] |
| Pyridoxine biosynthetic process | 4-Hydroxy-L-threonine phosphate dehydrogenase | PdxA | *pdx*A | 100 | 100 | 100 | | 329 | 12 | 100 | 98.3 | [39] |
| Pyridoxine biosynthetic process | Pyridoxal kinase | PdxK | *pdx*K | 100 | 100 | 100 | | 283 | 5 | 100 | 99.4 | [40] |
| Peptide catabolic process | Cytosol aminopeptidase | PepA | *pep*A | 100 | 100 | 100 | | 503 | 3 | 100 | 99.99 | [41] |
| Dephospho-rylation | Alkaline phosphatase | PhoA | *pho*A | 100 | 100 | 100 | | 471 | 16 | 100 | 98.5 | [42] |
| DNA repair (base-excision repair) | DNA polymerase I | PolA | *pol*A | 100 | 100 | 100 | | 928 | 13 | 100 | 99.4 | [43] |
| UMP biosynthetic process | Aspartate carbomoyltransferase | PyrB | *pyr*B | 100 | 100 | 100 | | 311 | 6 | 100 | 99.1 | [44] |
| UMP biosynthetic process | Dihydroorotase | PyrC | *pyr*C | 100 | 100 | 100 | | 348 | 8 | 100 | 99.0 | [45] |
| Pyrimidine nucleotide biosynthetic process | Aspartat carbamoyltransferase regulatory chain | PyrI | *pyr*I | 100 | 100 | 100 | | 153 | 2 | 100 | 99.96 | [46] |
| DNA repair | DNA helicase | RecQ | *rec*Q | 100 | 100 | 100 | | 609 | 9 | 100 | 98.6 | [47] |
| Rhamnose catabolic process | Rhamnulose-1-phosphate aldolase | RhaD | *rha*D | 100 | 100 | 100 | | 274 | 13 | 100 | 96.9 | [48] |
| Translation | Ribosomal protein L13 | RplM | *rpl*M | 100 | 100 | 100 | | 142 | 1 | 100 | 100 | [1] |
| Transcription | RNA polymerase β-subunit | RpoB | *rpo*B | 100 | 100 | 100 | | 1342 | 2 | 100 | 99.99 | [49] |
| Transcription | RNA polymerase subunit β’ | RpoC | *rpo*C | 100 | 100 | 100 | | 1407 | 3 | 100 | 99.98 | [50] |
| Regulation of translation (mRNA polyadenylation) | Ribosomal protein S15 | RpsO | *rps*O | 100 | 100 | 100 | | 89 | 2 | 100 | 99.9 | [1] |
| Translation (ribosomal small subunit assembly) | Ribosomal protein S16 | RpsP | *rps*P | 100 | 100 | 100 | | 82 | 1 | 100 | 100 | [1] |
| Translation (ribosomal small subunit assembly) | Ribosomal protein S17 | RpsQ | *rps*Q | 100 | 100 | 100 | | 84 | 1 | 100 | 100 | [1] |
| rRNA base methylation | Methyltransferase | RlmA | *rrm*A (*rlm*A) | 100 | 100 | 100 | | 269 | 13 | 100 | 99.1 | [51] |
| Superoxide metabolic process | Superoxide dismutase | SodC | *sod*C | 100 | 100 | 100 | | 173 | 5 | 100 | 99.5 | [52] |
| tRNA wobble adenosine to inosine editing | **t**RNA **a**denosine **d**eaminase | TadA | *tad*A | 100 | 100 | 100 | | 167 | 4 | 100 | 99.4 | [53] |
| L-serine/L-threonine catabolic process | Threonine dehydrogenase | Tdh | *tdh* | 100 | 100 | 100 | | 341 | 6 | 100 | 96.6 | [54] |
| Queuosine biosynthetic process | Queuine tRNA-ribosyltransferase | Tgt | *tgt* | 100 | 100 | 100 | | 375 | 4 | 100 | 99.6 | [55] |
| Regulation of translation (tRNA aminoacylation) | Threonyl-tRNA synthetase | ThrS | *thr*S | 100 | 100 | 100 | | 642 | 6 | 100 | 99.5 | [56] |
| Cellular response to antibiotic | Transketolase 1 | TktA | *tkt*A | 100 | 100 | 100 | | 663 | 7 | 100 | 98.8 | [57] |
| Pentose-phosphate shunt | Transketolase 2 | TktB | *tkt*B | 100 | 100 | 100 | | 667 | 14 | 100 | 99.0 | [1] |
| DNA topological change | DNA topoisomerase | TopA | *top*A | 100 | 100 | 100 | | 865 | 8 | 100 | 99.5 | [58] |
| Elongation | Translation elongation factor | Tsf | *tsf* | 100 | 100 | 100 | | 283 | 3 | 100 | 99.8 | [1] |
| DNA repair | Excision nuclease subunit A | UvrA | *uvr*A | 100 | 100 | 100 | | 940 | 5 | 100 | 99.98 | [59] |
| Mannitol metabolic process | D-mannonate oxidoreductase | UxuB | *uxu*B | 100 | 100 | 100 | | 486 | 15 | 100 | 98.1 | [60] |
| Bacterial-type flagellum-dependent swarming motility | Probable phosphatase | YcdX | *ycd*X | 100 | 100 | 100 | | 245 | 6 | 100 | 99.1 | [61] |

All 179 porcine commensal *E. coli* were screened with respect to the presence or absence of 69 factors involved in zinc homeostasis as zinc-binding metalloenzymes. Predicted amino acid sequence lengths were compared to those of *E. coli* K-12 MG1655 in order to check for putative premature stop codons or deletions affecting the putative function of the protein. None of the amino acid sequence variations (AV) was solely associated with a particular ZnCl_2_ MIC. Amino acid sequence variation among the isolate collection primarily reflects the isolate’s genetic backgrounds.

**Abbreviations:**

n, number of isolates; a, number of isolates with particular ZnCl_2_ MIC; L, length; AA, amino acid sequence; AV, number of amino acid sequence variants; C, amino acid sequence coverage with respect to reference protein in *E. coli* K-12 MG1655; I, maximum amino acid sequence identity among the 179 isolates;

**References**

[1] (Katayama et al., 2002); [2] (Vagelos and Larrabee, 1967); [3] (Mielecki and Grzesiuk, 2014); [4] (Gutheil et al., 1997); [5] (Sood et al., 1999); [6] (Agarwal et al., 2007); [7] (Kerff et al., 2010); [8] (Jacobs et al., 1994); [9] (Javid-Majd and Blanchard, 2000); [10] (Merlin et al., 2003); [11] (Xiang et al., 1997); [12] (Guilloton et al., 1992); [13] (Newberry et al., 2002); [14] (Lin et al., 1988); [15] (Loscha et al., 2004); [16] (Larsson et al., 1996); [17] (Kostelecky et al., 2006); [18] (Labbe et al., 2011); [19] (Auerbach et al., 2000); [20] (Dreyer and Schulz, 1996); [21] (Geeganage and Frey, 1999); [22] (O'Young et al., 2007); [23] (Banerjee et al., 2004)[24] (Maynes et al., 2000); [25] (Jaffe et al., 2002); [26] (Tomoyasu et al., 1995); [27] (Douglas et al., 2013) [28](Martí-Arbona et al., 2005); [29] (Lee and Poulter, 2006); [30] (Xu et al., 1994); [31] (Gattis et al., 2010); [32] (Chen et al., 2013); [33] (Gao et al., 2005); [34] (Hondorp and Matthews, 2004); [35] (Mayaux and Blanquet, 1981); [36] (Boiteux et al., 1990); [37] (Hall et al., 2007); [38] (Kropachev et al., 2006); [39] (Sivaraman et al., 2003); [40] (di Salvo et al., 2004); [41] (Straeter and Lipscomb, 1995); [42] (Sowadski et al., 1985); [43] (Springgate et al., 1973); [44] (Lipscomb and Kantrowitz, 2012); [45] (Lee et al., 2005); [46] (Rastogi et al., 1998); [47] (Liu et al., 2004); [48] (Kroemer et al., 2003); [49] (Alifano et al., 2015); [50] (Morse et al., 1996); [51] (Das et al., 2004); [52] (Pesce et al., 1997); [53] (Kim et al., 2006); [54] (Johnson et al., 1998); [55] (Chong et al., 1995); [56] (Caillet et al., 2007); [57] (Domain et al., 2007); [58] (Lu et al., 2011); [59] (Wang et al., 1994); [60] (Nguyen et al., 2008); [61](Teplyakov et al., 2003)

Agarwal, R., Burley, S.K., and Swaminathan, S. (2007). Structural Analysis of a Ternary Complex of Allantoate Amidohydrolase from Escherichia coli Reveals its Mechanics. *Journal of Molecular Biology* 368**,** 450-463.

Alifano, P., Palumbo, C., Pasanisi, D., and Tala, A. (2015). Rifampicin-resistance, rpoB polymorphism and RNA polymerase genetic engineering. *J Biotechnol* 202**,** 60-77.

Auerbach, G., Herrmann, A., Bracher, A., Bader, G., Gutlich, M., Fischer, M., Neukamm, M., Garrido-Franco, M., Richardson, J., Nar, H., Huber, R., and Bacher, A. (2000). Zinc plays a key role in human and bacterial GTP cyclohydrolase I. *Proceedings of the National Academy of Sciences of the United States of America* 97**,** 13567-13572.

Banerjee, R., Dubois, D.Y., Gauthier, J., Lin, S.-X., Roy, S., and Lapointe, J. (2004). The zinc-binding site of a class I aminoacyl-tRNA synthetase is a SWIM domain that modulates amino acid binding via the tRNA acceptor arm. *European Journal of Biochemistry* 271**,** 724-733.

Boiteux, S., O'connor, T.R., Lederer, F., Gouyette, A., and Laval, J. (1990). Homogeneous Escherichia coli FPG protein. A DNA glycosylase which excises imidazole ring-opened purines and nicks DNA at apurinic/apyrimidinic sites. *Journal of Biological Chemistry* 265**,** 3916-3922.

Caillet, J., Graffe, M., Eyermann, F., Romby, P., and Springer, M. (2007). Mutations in residues involved in zinc binding in the catalytic site of Escherichia coli threonyl-tRNA synthetase confer a dominant lethal phenotype. *J Bacteriol* 189**,** 6839-6848.

Chen, X., Boonyalai, N., Lau, C., Thipayang, S., Xu, Y., Wright, M., and Miller, A.D. (2013). Multiple catalytic activities of Escherichia coli lysyl-tRNA synthetase (LysU) are dissected by site-directed mutagenesis. *The FEBS Journal* 280**,** 102-114.

Chong, S., Curnow, A.W., Huston, T.J., and Garcia, G.A. (1995). tRNA-guanine transglycosylase from Escherichia coli is a zinc metalloprotein. Site-directed mutagenesis studies to identify the zinc ligands. *Biochemistry* 34**,** 3694-3701.

Das, K., Acton, T., Chiang, Y., Shih, L., Arnold, E., and Montelione, G.T. (2004). Crystal structure of RlmAI: implications for understanding the 23S rRNA G745/G748-methylation at the macrolide antibiotic-binding site. *Proceedings of the National Academy of Sciences of the United States of America* 101**,** 4041-4046.

Di Salvo, M.L., Hunt, S., and Schirch, V. (2004). Expression, purification, and kinetic constants for human and Escherichia coli pyridoxal kinases. *Protein Expression and Purification* 36**,** 300-306.

Domain, F., Bina, X.R., and Levy, S.B. (2007). Transketolase A, an enzyme in central metabolism, derepresses the marRAB multiple antibiotic resistance operon of Escherichia coli by interaction with MarR. *Mol Microbiol* 66**,** 383-394.

Douglas, C.D., Ngu, T.T., Kaluarachchi, H., and Zamble, D.B. (2013). Metal transfer within the Escherichia coli HypB-HypA complex of hydrogenase accessory proteins. *Biochemistry* 52**,** 6030-6039.

Dreyer, M.K., and Schulz, G.E. (1996). Catalytic Mechanism of the Metal-dependent Fuculose Aldolase fromEscherichia colias Derived from the Structure. *Journal of Molecular Biology* 259**,** 458-466.

Gao, H., Yu, Y., and Leary, J.A. (2005). Mechanism and Kinetics of Metalloenzyme Phosphomannose Isomerase:  Measurement of Dissociation Constants and Effect of Zinc Binding Using ESI-FTICR Mass Spectrometry. *Analytical Chemistry* 77**,** 5596-5603.

Gattis, S.G., Hernick, M., and Fierke, C.A. (2010). Active site metal ion in UDP-3-O-((R)-3-hydroxymyristoyl)-N-acetylglucosamine deacetylase (LpxC) switches between Fe(II) and Zn(II) depending on cellular conditions. *The Journal of biological chemistry* 285**,** 33788-33796.

Geeganage, S., and Frey, P.A. (1999). Significance of Metal Ions in Galactose-1-Phosphate Uridylyltransferase:  An Essential Structural Zinc and a Nonessential Structural Iron. *Biochemistry* 38**,** 13398-13406.

Guilloton, M.B., Korte, J.J., Lamblin, A.F., Fuchs, J.A., and Anderson, P.M. (1992). Carbonic anhydrase in Escherichia coli. A product of the cyn operon. *Journal of Biological Chemistry* 267**,** 3731-3734.

Gutheil, W.G., Kasimoglu, E., and Nicholson, P.C. (1997). Induction of Glutathione-Dependent Formaldehyde Dehydrogenase Activity in Escherichia coli and Hemophilus influenza. *Biochemical and Biophysical Research Communications* 238**,** 693-696.

Hall, R.S., Xiang, D.F., Xu, C., and Raushel, F.M. (2007). N-Acetyl-d-Glucosamine-6-Phosphate Deacetylase: Substrate Activation via a Single Divalent Metal Ion. *Biochemistry* 46**,** 7942-7952.

Hondorp, E.R., and Matthews, R.G. (2004). Oxidative stress inactivates cobalamin-independent methionine synthase (MetE) in Escherichia coli. *PLoS Biol* 2**,** e336.

Jacobs, C., Huang, L.J., Bartowsky, E., Normark, S., and Park, J.T. (1994). Bacterial cell wall recycling provides cytosolic muropeptides as effectors for beta-lactamase induction. *The EMBO journal* 13**,** 4684-4694.

Jaffe, E.K., Kervinen, J., Martins, J., Stauffer, F., Neier, R., Wlodawer, A., and Zdanov, A. (2002). Species-specific Inhibition of Porphobilinogen Synthase by 4-Oxosebacic Acid. *Journal of Biological Chemistry* 277**,** 19792-19799.

Javid-Majd, F., and Blanchard, J.S. (2000). Mechanistic Analysis of the argE-Encoded N-Acetylornithine Deacetylase. *Biochemistry* 39**,** 1285-1293.

Johnson, A.R., Chen, Y.-W., and Dekker, E.E. (1998). Investigation of a Catalytic Zinc Binding Site inEscherichia colil-Threonine Dehydrogenase by Site-Directed Mutagenesis of Cysteine-38. *Archives of Biochemistry and Biophysics* 358**,** 211-221.

Katayama, A., Tsujii, A., Wada, A., Nishino, T., and Ishihama, A. (2002). Systematic search for zinc-binding proteins inEscherichia coli. *European Journal of Biochemistry* 269**,** 2403-2413.

Kerff, F., Petrella, S., Mercier, F., Sauvage, E., Herman, R., Pennartz, A., Zervosen, A., Luxen, A., Frère, J.-M., Joris, B., and Charlier, P. (2010). Specific Structural Features of the N-Acetylmuramoyl-l-Alanine Amidase AmiD from Escherichia coli and Mechanistic Implications for Enzymes of This Family. *Journal of Molecular Biology* 397**,** 249-259.

Kim, J., Malashkevich, V., Roday, S., Lisbin, M., Schramm, V.L., and Almo, S.C. (2006). Structural and Kinetic Characterization of Escherichia coli TadA, the Wobble-Specific tRNA Deaminase. *Biochemistry* 45**,** 6407-6416.

Kostelecky, B., Pohl, E., Vogel, A., Schilling, O., and Meyer-Klaucke, W. (2006). The crystal structure of the zinc phosphodiesterase from Escherichia coli provides insight into function and cooperativity of tRNase Z-family proteins. *J Bacteriol* 188**,** 1607-1614.

Kroemer, M., Merkel, I., and Schulz, G.E. (2003). Structure and Catalytic Mechanism of l-Rhamnulose-1-phosphate Aldolase. *Biochemistry* 42**,** 10560-10568.

Kropachev, K.Y., Zharkov, D.O., and Grollman, A.P. (2006). Catalytic mechanism of Escherichia coli endonuclease VIII: roles of the intercalation loop and the zinc finger. *Biochemistry* 45**,** 12039-12049.

Labbe, G., De Groot, S., Rasmusson, T., Milojevic, G., Dmitrienko, G.I., and Guillemette, J.G. (2011). Evaluation of four microbial Class II fructose 1,6-bisphosphate aldolase enzymes for use as biocatalysts. *Protein Expr Purif* 80**,** 224-233.

Larsson, G., Nyman, P.O., and Kvassman, J.-O. (1996). Kinetic Characterization of dUTPase from Escherichia coli. *Journal of Biological Chemistry* 271**,** 24010-24016.

Lee, M., Chan, C.W., Mitchell Guss, J., Christopherson, R.I., and Maher, M.J. (2005). Dihydroorotase from Escherichia coli: Loop Movement and Cooperativity between Subunits. *Journal of Molecular Biology* 348**,** 523-533.

Lee, S., and Poulter, C.D. (2006). Escherichia coli Type I Isopentenyl Diphosphate Isomerase:  Structural and Catalytic Roles for Divalent Metals. *Journal of the American Chemical Society* 128**,** 11545-11550.

Lin, Y.K., Myhrman, R., Schrag, M.L., and Gelb, M.H. (1988). Bacterial N-succinyl-L-diaminopimelic acid desuccinylase. Purification, partial characterization, and substrate specificity. *J Biol Chem* 263**,** 1622-1627.

Lipscomb, W.N., and Kantrowitz, E.R. (2012). Structure and Mechanisms of Escherichia coli Aspartate Transcarbamoylase. *Accounts of Chemical Research* 45**,** 444-453.

Liu, J.L., Rigolet, P., Dou, S.-X., Wang, P.-Y., and Xi, X.G. (2004). The Zinc Finger Motif of Escherichia coli RecQ Is Implicated in Both DNA Binding and Protein Folding. *Journal of Biological Chemistry* 279**,** 42794-42802.

Loscha, K., Oakley, A.J., Bancia, B., Schaeffer, P.M., Prosselkov, P., Otting, G., Wilce, M.C.J., and Dixon, N.E. (2004). Expression, purification, crystallization, and NMR studies of the helicase interaction domain of Escherichia coli DnaG primase. *Protein Expression and Purification* 33**,** 304-310.

Lu, J., Wang, W., Tan, G., Landry, A.P., Yi, P., Si, F., Ren, Y., and Ding, H. (2011). Escherichia coli topoisomerase I is an iron and zinc binding protein. *Biometals : an international journal on the role of metal ions in biology, biochemistry, and medicine* 24**,** 729-736.

Martí-Arbona, R., Fresquet, V., Thoden, J.B., Davis, M.L., Holden, H.M., and Raushel, F.M. (2005). Mechanism of the Reaction Catalyzed by Isoaspartyl Dipeptidase from Escherichia coli. *Biochemistry* 44**,** 7115-7124.

Mayaux, J.F., and Blanquet, S. (1981). Binding of zinc to Escherichia coli phenylalanyl transfer ribonucleic acid synthetase. Comparison with other aminoacyl transfer ribonucleic acid synthetases. *Biochemistry* 20**,** 4647-4654.

Maynes, J.T., Yuan, R.G., and Snyder, F.F. (2000). Identification, expression, and characterization of Escherichia coli guanine deaminase. *Journal of bacteriology* 182**,** 4658-4660.

Merlin, C., Masters, M., Mcateer, S., and Coulson, A. (2003). Why Is Carbonic Anhydrase Essential to Escherichia coli? *Journal of Bacteriology* 185**,** 6415-6424.

Mielecki, D., and Grzesiuk, E. (2014). Ada response - a strategy for repair of alkylated DNA in bacteria. *FEMS Microbiol Lett* 355**,** 1-11.

Morse, R., Collins, M.D., Balsdon, J.T., Wallbanks, S., and Richardson, P.T. (1996). Nucleotide sequence of part of the rpoC gene encoding the β′ subunit of DNA-dependent RNA polymerase from some gram-positive bacteria and comparative amino acid sequence analysis. *Systematic and Applied Microbiology* 19**,** 150-157.

Newberry, K.J., Hou, Y.M., and Perona, J.J. (2002). Structural origins of amino acid selection without editing by cysteinyl-tRNA synthetase. *Embo j* 21**,** 2778-2787.

Nguyen, T.T., Brown, S., Fedorov, A.A., Fedorov, E.V., Babbitt, P.C., Almo, S.C., and Raushel, F.M. (2008). At the Periphery of the Amidohydrolase Superfamily:  Bh0493 from Bacillus halodurans Catalyzes the Isomerization of d-Galacturonate to d-Tagaturonate. *Biochemistry* 47**,** 1194-1206.

O'young, J., Sukdeo, N., and Honek, J.F. (2007). Escherichia coli glyoxalase II is a binuclear zinc-dependent metalloenzyme. *Arch Biochem Biophys* 459**,** 20-26.

Pesce, A., Capasso, C., Battistoni, A., Folcarelli, S., Rotilio, G., Desideri, A., and Bolognesi, M. (1997). Unique structural features of the monomeric Cu,Zn superoxide dismutase from Escherichia coli, revealed by X-ray crystallography11Edited by R. Huber. *Journal of Molecular Biology* 274**,** 408-420.

Rastogi, V.K., Swanson, R., Hartberg, Y.M., Wales, M.E., and Wild, J.R. (1998). Role of Allosteric:Zinc Interdomain Region of the Regulatory Subunit in the Allosteric Regulation of Aspartate Transcarbamoylase fromEscherichia coli. *Archives of Biochemistry and Biophysics* 354**,** 215-224.

Sivaraman, J., Li, Y., Banks, J., Cane, D.E., Matte, A., and Cygler, M. (2003). Crystal Structure of Escherichia coli PdxA, an Enzyme Involved in the Pyridoxal Phosphate Biosynthesis Pathway. *Journal of Biological Chemistry* 278**,** 43682-43690.

Sood, S.M., Wu, M.-X., Hill, K.a.W., and Slattery, C.W. (1999). Characterization of Zinc-Depleted Alanyl-tRNA Synthetase from Escherichia coli: Role of Zinc. *Archives of Biochemistry and Biophysics* 368**,** 380-384.

Sowadski, J.M., Handschumacher, M.D., Krishna Murthy, H.M., Foster, B.A., and Wyckoff, H.W. (1985). Refined structure of alkaline phosphatase from Escherichia coli at 2.8 Å resolution. *Journal of Molecular Biology* 186**,** 417-433.

Springgate, C.F., Mildvan, A.S., Abramson, R., Engle, J.L., and Loeb, L.A. (1973). Escherichia coli deoxyribonucleic acid polymerase I, a zinc metalloenzyme. Nuclear quadrupolar relaxation studies of the role of bound zinc. *J Biol Chem* 248**,** 5987-5993.

Straeter, N., and Lipscomb, W.N. (1995). Two-Metal Ion Mechanism of Bovine Lens Leucine Aminopeptidase: Active Site Solvent Structure and Binding Mode of L-Leucinal, a gem-Diolate Transition State Analog, by X-ray Crystallography. *Biochemistry* 34**,** 14792-14800.

Teplyakov, A., Obmolova, G., Khil Pavel, P., Howard Andrew, J., Camerini-Otero, R.D., and Gilliland Gary, L. (2003). Crystal structure of the Escherichia coli YcdX protein reveals a trinuclear zinc active site. *Proteins: Structure, Function, and Bioinformatics* 51**,** 315-318.

Tomoyasu, T., Gamer, J., Bukau, B., Kanemori, M., Mori, H., Rutman, A.J., Oppenheim, A.B., Yura, T., Yamanaka, K., and Niki, H. (1995). Escherichia coli FtsH is a membrane-bound, ATP-dependent protease which degrades the heat-shock transcription factor sigma 32. *The EMBO journal* 14**,** 2551-2560.

Vagelos, P.R., and Larrabee, A.R. (1967). Acyl Carrier Protein: IX. ACYL CARRIER PROTEIN HYDROLASE. *Journal of Biological Chemistry* 242**,** 1776-1781.

Wang, J., Mueller, K.L., and Grossman, L. (1994). A mutational study of the C-terminal zinc-finger motif of the Escherichia coli UvrA protein. *Journal of Biological Chemistry* 269**,** 10771-10775.

Xiang, S., Short, S.A., Wolfenden, R., and Carter, C.W. (1997). The Structure of the Cytidine Deaminase−Product Complex Provides Evidence for Efficient Proton Transfer and Ground-State Destabilization. *Biochemistry* 36**,** 4768-4774.

Xu, B., Trawick, B., Krudy, G.A., Phillips, R.M., Zhou, L., and Rosevear, P.R. (1994). Probing the metal binding sites of Escherichia coli isoleucyl-tRNA synthetase. *Biochemistry* 33**,** 398-402.
