# Supplementary material for: Effects of a Four-Week High-Dosage Zinc Oxide Supplemented Diet on Commensal Escherichia coli of Weaned Pigs
Source: Front Microbiol. 2019 Nov 28;10:2734. doi: 10.3389/fmicb.2019.02734 (PMC6892955; doi:10.3389/fmicb.2019.02734)
Supplement: Supplementary file 3 [file Table_3.docx]

| **Supplementary Table S3 Biocide-and heavy metal tolerance genes** | | | | | | | | | | |
| --- | --- | --- | --- | --- | --- | --- | --- | --- | --- | --- |
|  | |  |  | **Gene** | | | **Protein** | | | |
|  | |  |  | **ZnCl_2_-MICs (µg/ml)** | | |  | | | |
|  | |  |  | **128** | **256** | **512** |  |  |  |  |
| **Function** | | **Symbol** | **Gene** | **n=7** | **n=136** | **n=36** | **L** | **AV** | **C** | **I** |
|  | |  |  | **%** | **%** | **%** | **AA** | **n** | **%** | **%** |
|  | | **Biocide resistance chromosomally encoded genes** | | | | | | | |  |
| Acriflavine resistance protein A | | AcrA | *acr*A | 100 | 100 | 100 | 2 | 100 | 99.96 | 2 |
| Acriflavine resistance protein B | | AcrB | *acr*B | 100 | 100 | 100 | 4 | 100 | 99.9 | 4 |
| Acriflavine resistance protein D | | AcrD | *acr*D/  *yff*A | 100 | 100 | 100 | 13 | 100 | 99.9 | 13 |
| Acriflavine resistance protein E | | AcrE | *acr*E | 85.7 | 74.3 | 100 | 6 | 100 | 99.9 | 6 |
| Acriflavine resistance protein F | | AcrF | *acr*F | 85.7 | 71.3 | 94.4 | 16 | 100 | 99.6 | 16 |
| HTH-type potential regulator protein for the *acr*AB genes | | AcrR | *acr*R | 100 | 100 | 100 | 5 | 100 | 99.9 | 5 |
| Bicyclomycin resistance protein | | Bcr | *bcr* | 100 | 100 | 100 | 8 | 100 | 99.7 | 8 |
| Sensor histidine kinase, two-component regulatory system of CpxAR | | CpxA | *cpx*A | 100 | 100 | 100 | 4 | 100 | 99.9 | 4 |
| Response regulator, two-component regulatory system | | CpxR | *cpx*R | 100 | 100 | 100 | 2 | 100 | 99.9 | 2 |
| Multidrug export protein | | EmrA | *emr*A | 100 | 100 | 100 | 3 | 100 | 99.9 | 3 |
| Multidrug export protein | | EmrB | *emr*B | 100 | 100 | 100 | 4 | 100 | 99.9 | 4 |
| Multidrug resistance protein | | EmrD | *emr*D | 100 | 100 | 100 | 7 | 100 | 99.9 | 7 |
| Multidrug transporter | | EmrE | *emr*E/  *mvr*C | 28.7 | 40.4 | 30.6 | 3 | 100 | 99.4 | 3 |
| Probable Multidrug resistance protein | | EmrK | *emr*K | 100 | 100 | 100 | 9 | 100 | 99.5 | 9 |
| Transcriptional repressor | | EmrR | *emr*R | 100 | 100 | 100 | 3 | 100 | 99.9 | 3 |
| Multidrug resistance protein | | EmrY | *emr*Y | 100 | 100 | 100 | 6 | 100 | 99.7 | 6 |
| DNA-binding transcriptional activator | | EvgA | *evg*A | 100 | 100 | 100 | 2 | 100 | 99.9 | 2 |
| Sensor protein | | EvgS | *evg*S | 100 | 100 | 100 | 9 | 100 | 99.1 | 9 |
| Enoyl-[acyl-carrier protein] reductase | | FabI | *fab*I | 100 | 100 | 100 | 3 | 100 | 99.9 | 3 |
| Glutamate decarboxylase α | | GadA | *gad*A | 100 | 26.5 | 25.0 | 3 | 100 | 99.9 | 3 |
| Glutamate decarboxylase β | | GadB | *gad*B | 100 | 26.5 | 25.0 | 3 | 100 | 99.9 | 3 |
| Probable glutamate/γ-aminobutyrate antiporter | | GadC | *gad*C | 100 | 100 | 100 | 6 | 100 | 99.9 | 6 |
| Transcriptional regulator | | GadE | *gad*E | 100 | 100 | 100 | 1 | 100 | 100 | 1 |
| HTH-type transcriptional regulator | | GadW | *gad*W | 100 | 100 | 100 | 7 | 100 | 99.0 | 7 |
| HTH-type transcriptional regulator | | GadX | *gad*X | 100 | 100 | 100 | 9 | 100 | 98.6 | 9 |
| Acid stress chaperone | | HdeB | *hde*B | 100 | 100 | 100 | 2 | 100 | 99.97 | 2 |
| Small heat shock protein | | IbpA | *ibp*A | 100 | 100 | 100 | 1 | 100 | 100 | 1 |
| Small heat shock protein | | IbpB | *ibp*B | 100 | 100 | 100 | 3 | 100 | 99.9 | 3 |
| Acetate operon repressor | | IclR | *icl*R | 100 | 100 | 100 | 4 | 100 | 99.9 | 4 |
| Transcriptional activator | | MarA | *mar*A | 100 | 100 | 100 | 1 | 100 | 99.6 | 1 |
| Multiple antibiotic resistance protein | | MarR | *mar*R | 100 | 100 | 100 | 4 | 100 | 99.2 | 4 |
| Multidrug transporter | | MdfA | *mdf*A | 100 | 100 | 100 | 12 | 100 | 99.5 | 12 |
| Multidrug resistance protein | | MdtA | *mdt*A | 100 | 100 | 100 | 19 | 100 | 99.2 | 19 |
| Multidrug resistance protein | | MdtB | *mdt*B | 100 | 100 | 100 | 23 | 100 | 99.6 | 23 |
| Multidrug resistance protein | | MdtC | *mdt*C | 100 | 100 | 100 | 17 | 100 | 99.7 | 17 |
| Multidrug resistance protein | | MdtE | *mdt*E | 100 | 100 | 100 | 6 | 100 | 99.9 | 6 |
| Multidrug resistance protein | | MdtF | *mdt*F | 100 | 100 | 100 | 8 | 100 | 99.2 | 8 |
| Multidrug resistance protein | | MdtG | *mdt*G | 100 | 100 | 100 | 9 | 100 | 99.9 | 9 |
| Spermidine export protein | | MdtI | *mdt*I | 100 | 100 | 100 | 7 | 100 | 99.6 | 7 |
| Spermidine export protein | | MdtJ | *mdt*J | 100 | 100 | 100 | 5 | 100 | 99.7 | 5 |
| Multidrug resistance protein | | MdtK | *mdt*K | 100 | 100 | 100 | 7 | 100 | 99.8 | 7 |
| Multidrug resistance protein | | MdtM | *mdt*M | 100 | 96.3 | 97.2 | 13 | 100 | 99.1 | 13 |
| Multidrug resistance protein | | MdtN | *mdt*N | 100 | 100 | 100 | 15 | 100 | 99.5 | 15 |
| Organic solvent tolerance protein | | OstA/LptD | *ost*A | 100 | 100 | 100 | 9 | 100 | 99.9 | 9 |
| Activator of hydrogen peroxide-inducible genes | | OxyR | *oxy*Rkp | 100 | 100 | 100 | 1 | 100 | 99.96 | 1 |
| Phosphate regulon transcriptional regulatory protein | | PhoB | *pho*B | 100 | 100 | 100 | 5 | 100 | 99.6 | 5 |
| RNA polymerase sigma factor | | RpoS | *rpo*S | 100 | 100 | 100 | 1 | 100 | 99.99 | 1 |
| Redox-sensitive transcriptional activator | | SoxR | *sox*R | 100 | 100 | 100 | 4 | 100 | 99.8 | 4 |
| Outer membrane protein | | TolC | *tol*C | 100 | 100 | 100 | 5 | 100 | 99.9 | 5 |
| Stress protein | | YdeI | *yde*I | 100 | 97.1 | 97.2 | 6 | 100 | 99.3 | 6 |
| HTH-type transcriptional regulator | | YdeO | *yde*O | 100 | 100 | 100 | 9 | 100 | 99.3 | 9 |
| Acid resistance protein | | YdeP | *yde*P | 100 | 100 | 100 | 19 | 100 | 99.4 | 19 |
| **Biocide resistance plasmid-located genes** | | | | | | | | | | |
| Disinfectant resistance protein | QacEΔ1 | | *qac*EΔ1 | 0 | 8.1 | 52.8 | 115 | 1 | 100 | 100 |
| Quaternary ammonium compound-resistance protein | QacL | | *qac*L | 0 | 9.6 | 52.8 | 110 | 1 | 100 | 100 |
| Quaternary ammonium compound-resistance protein | SugE | | *sug*E | 100 | 100 | 100 | 105 | 5 | 100 | 99.8 |
| Probable two-component-system connector protein | YmgB/  AriR | | *ymg*B | 100 | 94.1 | 100 | 88 | 4 | 100 | 99.7 |
| **Heavy metal resistance chromosomally encoded genes** | | | | | | | | | | |
| Acriflavine resistance protein | | AcrD | *acr*D | 100 | 100 | 100 | 1037 | 14 | 100 | 99.8 |
| Arsenite inducible repressor | | ArsR | *ars*R | 100 | 100 | 100 | 117 | 4 | 100 | 96.8 |
| Multiple stress resistance protein | | BhsA | *bhs*A/  *ycf*R | 100 | 100 | 100 | 85 | 1 | 100 | 100 |
| HTH-type transcriptional repressor | | ComR | *com*R/  *ycf*Q | 100 | 100 | 100 | 210 | 5 | 100 | 99.7 |
| Magnesium transport protein | | CorA | *cor*A | 100 | 100 | 100 | 316 | 3 | 100 | 99.98 |
| Magnesium-transporting P-type-1-ATPase | | CorB | *cor*B | 100 | 100 | 100 | 227 | 4 | 100 | 99.9 |
| Magnesium and cobalt efflux protein | | CorC | *cor*C | 100 | 100 | 100 | 292 | 1 | 100 | 99.97 |
| Unknown protein involved in Magnesium transport | | CorD | *cor*D | 100 | 100 | 100 | 125 | 1 | 100 | 99.9 |
| Copper efflux oxidase | | CueO | *cue*O | 100 | 100 | 100 | 516 | 13 | 100 | 99.4 |
| HTH-type transcriptional regulator | | CueR | *cue*R/  *ybb*I | 100 | 100 | 100 | 135 | 3 | 100 | 99.9 |
| Sensor kinase | | CusS | *cus*S | 100 | 100 | 100 | 480 | 13 | 100 | 98.7 |
| Divalent-cation tolerance protein | | CutA | *cut*A | 100 | 100 | 100 | 112 | 5 | 100 | 99.7 |
| Copper homeostasis protein | | CutC | *cut*C | 100 | 100 | 100 | 248 | 14 | 100 | 99.0 |
| Apolipoprotein N-acyltransferase | | CutE | *cut*E | 100 | 100 | 100 | 512 | 12 | 100 | 99.2 |
| Copper homeostasis protein | | CutF | *cut*F/  *nlp*E | 100 | 100 | 100 | 236 | 9 | 100 | 99.0 |
| Disulfide oxidoreductase | | DsbA | *dsb*A | 100 | 100 | 100 | 208 | 5 | 100 | 99.9 |
| Disulfide oxidoreductase | | DsbB | *dsb*B | 100 | 100 | 100 | 176 | 5 | 100 | 99.6 |
| Disulfide isomerase | | DsbC | *dsb*C | 100 | 100 | 100 | 236 | 7 | 100 | 99.5 |
| Fe(^3+^) dicitrate transport system permease protein | | FecD | *fec*D | 85.7 | 60.3 | 52.8 | 318 | 2 | 100 | 99.98 |
| Fe(^3+^) dicitrate transport ATP-binding protein | | FecE | *fec*E | 85.7 | 60.3 | 52.8 | 255 | 2 | 100 | 99.98 |
| Probable iron export ATP binding component subunit | | FetA | *fet*A/  *ybb*L | 100 | 100 | 100 | 255 | 10 | 100 | 99.5 |
| Probable iron export permease protein | | FetB | *fet*B/  *ybb*M | 100 | 100 | 100 | 259 | 6 | 100 | 99.7 |
| Iron-efflux transporter | | FieF | *fie*F, *yiip* | 100 | 100 | 100 | 300 | 4 | 100 | 99.9 |
| Glycerol uptake facilitator protein | | GlpF | *glp*F | 100 | 100 | 100 | 281 | 2 | 100 | 99.99 |
| Magnesium-transporting P-type 1 ATPase | | MgtA | *mgt*A | 100 | 100 | 100 | 902 | 9 | 100 | 99.7 |
| Divalent metal cation transporter | | MntH | *mnt*H/  *yfe*P | 100 | 100 | 100 | 412 | 6 | 100 | 99.9 |
| Manganese efflux pump | | MntP | *mnt*P | 100 | 100 | 100 | 188 | 2 | 100 | 99.9 |
| Manganese transport regulator | | MntR | *mnt*R | 100 | 100 | 100 | 155 | 1 | 100 | 100 |
| Periplasmic molybdate-binding ABC transporter protein | | ModA | *mod*A | 100 | 100 | 100 | 257 | 12 | 100 | 98.7 |
| Molybdenum ABC transporter permease protein | | ModB | *mod*B | 100 | 100 | 100 | 233 | 4 | 100 | 99.9 |
| Molybdenum import ATP-binding protein | | ModC | *mod*C | 100 | 100 | 100 | 253 | 12 | 100 | 99.5 |
| Transcriptional regulator | | ModE | *mod*E | 100 | 100 | 100 | 262 | 6 | 100 | 99.6 |
| Oxygen-insensitive nitroreductase | | NfsA | *nfs*A | 100 | 100 | 100 | 240 | 8 | 100 | 94.4 |
| Nickel-binding periplasmic protein | | NikA | *nik*A | 100 | 100 | 100 | 524 | 8 | 100 | 99.7 |
| Nickel transport system permease protein | | NikB | *nik*B | 100 | 100 | 100 | 314 | 8 | 100 | 99.9 |
| Nickel transport system permease protein | | NikC | *nik*C | 100 | 100 | 100 | 277 | 8 | 100 | 99.7 |
| Nickel import ATP-binding protein | | NikD | *nik*D | 100 | 100 | 100 | 254 | 10 | 100 | 99.6 |
| Nickel import ATP-binding protein | | NikE | *nik*E | 100 | 100 | 100 | 268 | 8 | 100 | 99.0 |
| Nickel-responsive regulator | | NikR | *nik*R | 100 | 100 | 100 | 133 | 2 | 100 | 99.97 |
| Low-affinity inorganic phosphate transporter 1 | | PitA | *pit*A | 100 | 100 | 100 | 499 | 3 | 100 | 99.99 |
| Phosphate transport system permease protein | | PstA | *pst*A | 100 | 100 | 100 | 296 | 8 | 100 | 99.7 |
| Phosphate transport system ATPase | | PstB | *pst*B | 100 | 100 | 100 | 257 | 2 | 100 | 99.97 |
| Phosphate transport system integral membrane permease protein | | PstC | *pst*C | 100 | 100 | 100 | 319 | 3 | 100 | 99.9 |
| Phosphate transport system periplasmic binding protein | | PstS | *pst*S | 100 | 100 | 100 | 346 | 5 | 100 | 99.9 |
| Nickel/cobalt efflux system protein | | RcnA | *rcn*A | 100 | 100 | 100 | 274 | 16 | 100 | 96.1 |
| Nickel/cobalt homeostasis protein | | RcnB | *rcn*B | 100 | 100 | 100 | 112 | 4 | 100 | 99.5 |
| Transcriptional repressor | | RcnR | *rcn*R/  *yoh*L | 100 | 100 | 100 | 90 | 2 | 100 | 99.96 |
| Fe^3+^-Yersiniabactin uptake transporter | | YbtP | *ybt*P | 100 | 100 | 100 | 600 | 7 | 100 | 99.6 |
| Permease and ATP-binding protein of Yersiniabactin-iron ABC transport | | YbtQ | *ybt*Q | 100 | 100 | 100 | 600 | 5 | 100 | 99.7 |
| NADPH-dependent ferric-chelate reductase | | YqjH | *yqj*H | 100 | 99.3 | 100 | 254 | 11 | 100 | 99.2 |
| **Heavy metal reistance plasmid encoded genes** | | | | | | | | | | |
| Arsenical pump-driving ATPase | | ArsA | *ars*A | 14.3 | 25.7 | 0 | 583 | 1 | 100 | 99.2 |
| Arsenical pump membrane protein | | ArsB | *ars*B | 85.7 | 93.4 | 91.7 | 429 | 3 | 100 | 98.0 |
| Arsenate reductase | | ArsC | *ars*C | 100 | 100 | 100 | 141 | 11 | 100 | 96.7 |
| Arsenical resistance operon trans-acting repressor | | ArsD | *ars*D | 14.3 | 25.7 | 0 | 120 | 1 | 100 | 99.2 |
| Arsenical resistance operon repressor | | ArsR | *ars*R | 85.7 | 79.4 | 80.6 | 117 | 2 | 100 | 91.5 |
| Cation efflux system protein | | CusA | *cus*A | 100 | 100 | 100 | 1047 | 16 | 100 | 99.0 |
| Cation efflux system protein | | CusB | *cus*B | 100 | 100 | 100 | 407 | 11 | 100 | 99.2 |
| Cation efflux system protein | | CusC | *cus*C | 100 | 100 | 100 | 457 | 15 | 100 | 99.2 |
| Cation efflux system periplasmic protein | | CusF | *cus*F | 100 | 100 | 100 | 110 | 11 | 100 | 98.5 |
| Transcriptional regulatory protein | | CusR | *cus*R | 100 | 100 | 100 | 227 | 4 | 100 | 99.8 |
| mercuric resistance operon regulator protein | | MerR | *mer*R | 0 | 8.1 | 52.8 | 135 | 1 | 100 | 100 |
| Mercuric ion transport protein | | MerT | *mer*T | 0 | 8.1 | 52.8 | 116 | 2 | 100 | 99.2 |
| Copper resistance protein | | PcoA | *pco*A | 14.3 | 25.7 | 0 | 605 | 2 | 100 | 99.98 |
| Copper resistance protein | | PcoB | *pco*B | 14.3 | 25.7 | 0 | 296 | 1 | 100 | 100 |
| Copper resistance protein | | PcoC | *pco*C | 14.3 | 25.7 | 0 | 126 | 2 | 100 | 99.96 |
| Copper resistance protein | | PcoD | *pco*D | 14.3 | 25.7 | 0 | 309 | 1 | 100 | 100 |
| Copper-binding protein | | PcoE | *pco*E | 14.3 | 25.7 | 0 | 144 | 2 | 100 | 99.6 |
| Transcriptional regulatory protein | | PcoR | *pco*R | 14.3 | 25.7 | 0 | 226 | 1 | 100 | 100 |
| Sensor protien | | PcoS | *pco*S | 14.3 | 25.7 | 0 | 466 | 2 | 100 | 99.97 |
| Inner membrane chemiosmotic, cation/proton antiporter | | SilA | *sil*A | 14.3 | 25.7 | 0 | 1048 | 2 | 100 | 99.9 |
| Sil cation-efflux system membrane fusion protein | | SilB | *sil*B | 14.3 | 25.7 | 0 | 430 | 2 | 100 | 99.8 |
| Sil cation-efflux system outer membran lipoprotein | | SilC | *sil*C | 14.3 | 25.7 | 0 | 461 | 2 | 100 | 99.9 |
| Sil cation-efflux system binding protein | | SilE | *sil*E | 14.3 | 25.7 | 0 | 143 | 1 | 100 | 100 |
| Sil cation-efflux system periplasmic chaperone | | SilF | *sil*F | 14.3 | 25.7 | 0 | 96 | 2 | 100 | 99.8 |
| Sil cation efflux system P-type ATPase | | SilP | *sil*P | 14.3 | 25.7 | 0 | 824 | 2 | 100 | 99.7 |
| Transcriptional regulator | | SilR | *sil*R | 14.3 | 25.7 | 0 | 228 | 2 | 100 | 99.6 |
| Membrane sensor kinase | | SilS | *sil*S | 14.3 | 25.7 | 0 | 497 | 2 | 100 | 99.6 |
| Tellurite resistance protein | | TerB | *ter*B | 0 | 0 | 52.8 | 122 | 2 | 100 | 99.2 |
| Tellurite resistance protein | | TerC | *ter*C | 0 | 0 | 52.8 | 346 | 2 | 100 | 99.2 |
| Tellurite resistance protein | | TerD | *ter*D | 0 | 0 | 52.8 | 192 | 2 | 100 | 99.2 |
| Tellurite resistance protein | | TerW | *ter*W | 0 | 0 | 52.8 | 155 | 2 | 100 | 99.2 |
| Tellurite resistance protein | | TerZ | *ter*Z | 0 | 0 | 52.8 | 193 | 2 | 100 | 99.2 |
| bacterial oligonucleotide/oligosaccharide binding fold (BOF) family Protein | | YgiW | *ygi*W | 100 | 100 | 100 | 130 | 2 | 100 | 99.95 |
| **Genes that have both biocide resistance and heavy metal tolerance potential** | | | | | | | | | | |
| Cation/acetate symporter | | ActP | *act*P | 100 | 100 | 100 | 549 | 5 | 100 | 99.9 |
| Transcriptional regulatory proten | | BaeR | *bae*R | 100 | 100 | 100 | 240 | 9 | 100 | 99.3 |
| Signal transductin histidine-protein kinase | | BaeS | *bae*S | 100 | 100 | 100 | 467 | 12 | 100 | 99.2 |
| Probable iron export ATP-binding protein | | FetA | *fet*A | 100 | 100 | 100 | 225 | 10 | 100 | 99.5 |
| Probable iron export permease protein | | FetB | *fet*B | 100 | 100 | 100 | 259 | 7 | 100 | 99.7 |
| Glycerol uptake facilitator protein | | GlpF | *glp*F | 100 | 100 | 100 | 281 | 2 | 100 | 99.99 |
| Mercury resistance operon negative regulator | | MerR2 | *mer*R2 | 0.0 | 8.1 | 52.8 | 144 | 1 | 100 | 99.5 |
| Probable manganese efflux pump | | MntP | *mnt*P | 100 | 100 | 100 | 188 | 3 | 100 | 99.9 |
| Right origin-binding protein | | RobA | *rob*A | 100 | 100 | 100 | 289 | 3 | 100 | 99.9 |
| Mn-dependent superoxide dismutase | | SodA | *sod*A | 100 | 100 | 100 | 206 | 7 | 100 | 99.6 |
| Fe-dependent superoxide dismutase | | SodB | *sod*B | 100 | 100 | 100 | 193 | 2 | 100 | 99.98 |
| Regulatory protein | | SoxS | *sox*S | 100 | 100 | 100 | 107 | 1 | 100 | 100 |
| Tellurite resistance protein | | TehA | *teh*A | 100 | 100 | 100 | 330 | 8 | 100 | 99.7 |
| S-adenosyl-L-methionine dependent methyltransferase | | TehB | *teh*B | 100 | 100 | 100 | 197 | 12 | 100 | 99.3 |
| Stress protein | | YchH | *ych*H | 100 | 100 | 100 | 92 | 1 | 100 | 100 |
| bacterial oligonucleotide/oligosaccharide binding fold (BOF) family Protein | | YgiW | *ygi*W | 100 | 100 | 100 | 130 | 2 | 100 | 99.95 |
| Outer membrane protein | | YhcN | *yhc*N | 100 | 100 | 100 | 87 | 2 | 100 | 99.8 |
| Acyl carrier protein phosphodiesterase | | YieF | *yie*F | 100 | 100 | 100 | 188 | 12 | 100 | 99.5 |
| Stress response protein | | YjaA | *yja*A | 85.7 | 63.2 | 61.1 | 127 | 5 | 100 | 98.9 |
| Stress protein | | YodD | *yod*D | 100 | 100 | 100 | 75 | 1 | 100 | 100 |
| Cation/acetate symporter | | ActP | *act*P | 100 | 100 | 100 | 549 | 5 | 100 | 99.9 |
| Transcriptional regulatory protein | | BaeR | *bae*R | 100 | 100 | 100 | 240 | 9 | 100 | 99.3 |
| Signal transduction histidine-protein kinase | | BaeS | *bae*S | 100 | 100 | 100 | 467 | 12 | 100 | 99.2 |
| Probable iron export ATP-binding protein | | FetA | *fet*A | 100 | 100 | 100 | 225 | 10 | 100 | 99.5 |
| Probable iron export permease protein | | FetB | *fet*B | 100 | 100 | 100 | 259 | 7 | 100 | 99.7 |
| Glycerol uptake facilitator protein | | GlpF | *glp*F | 100 | 100 | 100 | 281 | 2 | 100 | 99.99 |
| Mercury resistance operon negative regulator | | MerR2 | *mer*R2 | 0.0 | 8.1 | 52.8 | 144 | 1 | 100 | 99.5 |
| Probable manganese efflux pump | | MntP | *mnt*P | 100 | 100 | 100 | 188 | 3 | 100 | 99.9 |
| Right origin-binding protein | | RobA | *rob*A | 100 | 100 | 100 | 289 | 3 | 100 | 99.9 |
| Mn-dependent superoxide dismutase | | SodA | *sod*A | 100 | 100 | 100 | 206 | 7 | 100 | 99.6 |
| Fe-dependent superoxide dismutase | | SodB | *sod*B | 100 | 100 | 100 | 193 | 2 | 100 | 99.98 |
| Regulatory protein | | SoxS | *sox*S | 100 | 100 | 100 | 107 | 1 | 100 | 100 |
| Tellurite resistance protein | | TehA | *teh*A | 100 | 100 | 100 | 330 | 8 | 100 | 99.7 |
| S-adenosyl-L-methionine dependent methyltransferase | | TehB | *teh*B | 100 | 100 | 100 | 197 | 12 | 100 | 99.3 |
| Stress protein | | YchH | *ych*H | 100 | 100 | 100 | 92 | 1 | 100 | 100 |
| bacterial oligonucleotide/oligosaccharide binding fold (BOF) family Protein | | YgiW | *ygi*W | 100 | 100 | 100 | 130 | 2 | 100 | 99.95 |
| Outer membrane protein | | YhcN | *yhc*N | 100 | 100 | 100 | 87 | 2 | 100 | 99.8 |
| Acyl carrier protein phosphodiesterase | | YieF | *yie*F | 100 | 100 | 100 | 188 | 12 | 100 | 99.5 |
| Stress response protein | | YjaA | *yja*A | 85.7 | 63.2 | 61.1 | 127 | 5 | 100 | 98.9 |
| Stress protein | | YodD | *yod*D | 100 | 100 | 100 | 75 | 1 | 100 | 100 |

All 179 porcine commensal *E. coli* were screened with respect to the presence or absence of 203 factors described by Pal et al. (2013) particularly known to be associated with increased tolerance or even resistance toward different biocides or heavy metals. Predicted amino acid sequence lengths were compared to those of *E. coli* K-12 MG1655 in order to check for putative premature stop codons or deletions affecting the putative function of the protein. None of the amino acid sequence variations (AV) was solely associated with a particular ZnCl_2_ MIC. Amino acid sequence variation among the isolate collection primarily reflects the isolate’s genetic backgrounds.

**Abbreviation**

n, number of isolates; L, length; AA, amino acid; AV, number of amino acid sequence variants; C, amino acid sequence coverage with respect to BacMet data base (Pal et al., 2013); I, maximum amino acid sequence identity among the 179 isolates ; REF, references

Pal, C., Bengtsson-Palme, J., Rensing, C., Kristiansson, E., and Larsson, D.G.J. (2013). BacMet: antibacterial biocide and metal resistance genes database. *Nucleic Acids Research* 42**,** D737-D743.
