# Supplementary material for: Effects of a Four-Week High-Dosage Zinc Oxide Supplemented Diet on Commensal Escherichia coli of Weaned Pigs
Source: Front Microbiol. 2019 Nov 28;10:2734. doi: 10.3389/fmicb.2019.02734 (PMC6892955; doi:10.3389/fmicb.2019.02734)
Supplement: Supplementary file 5 [file Table_5.docx]

| **Factor** | **Regression**  **coefficient** | **p-value** | **95% CI** |
| --- | --- | --- | --- |
|  |  |  |  |
| **Intercept** | 8.212 | <0.001 | 7.747-8.677 |
| **Mucosa** | 0.005 | 0.948 | -0.140-0.150 |
| **Digesta** | 0.062 | 0.392 | -0.08-0.203 |
| **Feces** | 0^b^ | 0.640^a^ | . |
| **HZG** | 0^b^ | . | . |
| **CG** | -0.326 | **0.011** | -0.566-(-0.086) |
| **lg2 ACR MIC** | 0.017 | 0.746 | -0.086-0.119 |

**Supplementary Table 5A.** Results of mixed linear regression model examining the influence of lg2 acridine MICs, feeding group and sample site on lg2 ZnCl_2_ MIC (dependent factor) for 179 *E. coli* with pig as random variable

Abbreviations: HZG, high zinc group; CG, control group; CI, Confidence interval; ACR, acridine.

^a^ Global p-value determined for three sampling sites (mucosa, digesta, feces). ^b^ This parameter is set to zero because it is redundant. Bold numbers indicate a significant p-value.

**Supplementary Table 5B.** Results of mixed linear regression model examining the influence of lg2 silver nitrate MICs, feeding group and sample site on lg2 ZnCl_2_ MIC (dependent factor) for 179 *E. coli* with pig as random variable

| **Factor** | **Regression**  **coefficient** | **p-value** | **95% CI** |
| --- | --- | --- | --- |
|  |  |  |  |
| **Intercept** | 8.284 | <0.001 | 8.072-8.497 |
| **Mucosa** | 0.004 | 0.955 | -0.141-0.149 |
| **Digesta** | 0.061 | 0.397 | -0.081-0.203 |
| **Feces** | 0^b^ | 0.643^a^ | . |
| **HZG** | 0^b^ | . | . |
| **CG** | -0.331 | **0.010** | -0.569-(-0.093) |
| **lg2 SIL MIC** | -0.002 | 0.979 | -0.139-0.136 |

Abbreviations: HZG, high zinc group; CG, control group; CI, Confidence interval; SIL, silver nitrate.

^a^ Global p-value determined for three sampling sites (mucosa, digesta, feces). ^b^ This parameter is set to zero because it is redundant. Bold numbers indicate a significant p-value.

**Supplementary Table 5C.** Results of mixed linear regression model examining the influence of lg2 chlorhexidine MICs, feeding group and sample site on lg2 ZnCl_2_ MIC (dependent factor) for 179 *E. coli* with pig as random variable

| **Factor** | **Regression coefficient** | **p-value** | **95% CI** |
| --- | --- | --- | --- |
|  |  |  |  |
| **Intercept** | 8.118 | <0.001 | 7.900-8.336 |
| **Mucosa** | 0.046 | 0.535 | -0.101-0.194 |
| **Digesta** | 0.109 | 0.143 | -0.037-0.255 |
| **Feces** | 0^b^ | . | . |
| **HZG** | 0^b^ | . | . |
| **CG** | -0.187 | 0.137 | -0.437-0.063 |
| **lg2 CHX MIC** | -0.193 | **0.022** | -0.358-(-0.028) |
| **HZG*lg2 CHX MIC** | 0^b^ | . | . |
| **CG*lg2 CHX MIC** | 0.213 | **0.047** | 0.003-0.424 |

Abbreviations: HZG, high zinc group; CG, control group; CI, Confidence interval; CHX, Chlorhexidine.

^a^ Global p-value determined for three sampling sites (mucosa, digesta, feces). ^b^ This parameter is set to zero because it is redundant. Bold numbers indicate a significant p-value.
